# Epigenetic LOCK chromatin links H3K9 modifications to ATM–γH2AX signaling in HIV-Tat and opioid neuroinflammation

**DOI:** 10.1038/s41419-026-09146-6

**Published:** 2026-08-05

**Authors:** Karthick Chennakesavan, Malar Samikkannu, Haylee R. Hammond, Shainnel O. Eans, Marc J. Kaufman, Jay P. McLaughlin, Thangavel Samikkannu

**Affiliations:** 1 Department of Pharmaceutical Sciences, Irma Lerma Rangel College of Pharmacy, Texas A&M University, College Station, TX USA; 2 Department of Pharmacodynamics, College of Pharmacy, University of Florida, Gainesville, FL USA; 3 McLean Imaging Center, McLean Hospital/Harvard Medical School, Belmont, MA USA

**Keywords:** Epigenetics and behaviour, Chromatin remodelling

## Abstract

HIV-Tat and morphine synergistically exacerbate neuroinflammation and DNA damage, yet the mechanisms linking the DNA damage response (DDR), chromatin remodeling, and innate immune activation remain poorly understood. Here, we investigated how HIV-Tat and morphine alter DNA repair dynamics and cGAS–STING–AIM2 inflammasome signaling in microglia and cortical tissue and evaluated the efficacy of dimethyl fumarate (DMF) in mitigating these effects. HMC3 microglial cells were exposed to HIV-Tat (50 ng/mL) and morphine (10 µM) for 24 h or 5 days, with H2A.X knockdown performed prior to treatment. In vivo, doxycycline (DOX)-induced Tat transgenic mice received escalating morphine doses for 4 days, followed by DMF (30 mg/kg, i.p.) for 7 days. Postmortem frontal cortical tissues from HIV-positive and matched HIV-negative donors were analyzed for validation. Behavioral testing, immunostaining, qPCR, and Western blotting assessed DDR markers, KAT5 acetylation, histone modifications, and innate immune mediators. HIV-Tat and morphine significantly enhanced DDR activation in vitro and in vivo, as indicated by increased ATM and γH2AX and elevated KAT5 acetylation. Large Organized Chromatin Lysine (K) domains (LOCK) displayed reduced H3K9me3 and increased H3K9ac, reflecting chromatin relaxation and disrupted epigenetic control over DDR and inflammation. DOX+Morphine treatment upregulated ERCC1 and ERCC8 but downregulated ERCC2, ERCC3, and ERCC6, demonstrating impaired nucleotide excision repair, and these changes were closely associated with cGAS–STING activation and AIM2/NLRP3 inflammasome induction. H2A.X knockdown or DMF treatment suppressed ATM phosphorylation, restored H3K9me3, normalized ERCC expression, and attenuated inflammasome activation. DMF also rescued HIV-Tat ± morphine-induced deficits in novel object recognition. Postmortem cortical tissue confirmed γH2AX accumulation, reduced ERCC2/ERCC3, and altered histone signatures consistent with experimental findings. Together, these results demonstrate that HIV-Tat and morphine cooperatively disrupt LOCK chromatin and DDR through the ATM/γH2AX axis, and that DMF reverses these molecular and functional abnormalities, highlighting its therapeutic potential in mitigating HIV- and opioid-induced neuroinflammation and cognitive decline.

**Figure:**
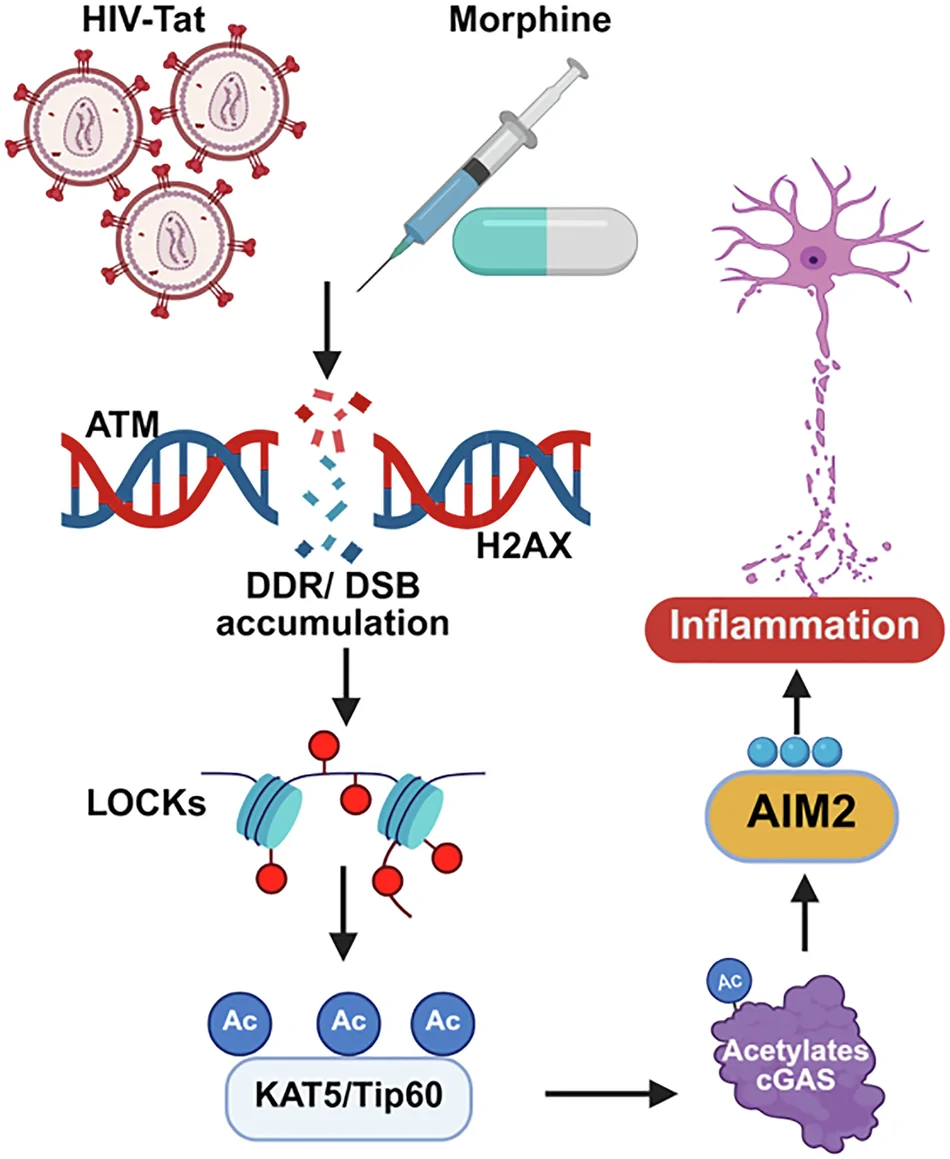

## Highlights

- HIV-Tat protein and morphine induce DNA damage, dysregulated ERCC repair, and LOCK chromatin remodeling in vivo and in vitro.
- KAT5/Tip60 acetylation and LOCK relaxation activate the cGAS–STING and AIM2/NLRP3 inflammasome signaling pathways.
- H2A.X knockdown reduces DDR, LOCK alterations, and inflammasome activation in HMC3 cells.
- Dimethyl fumarate restores ERCCs balance, normalizes LOCK-associated chromatin marks, and suppresses DDR and innate immune activation, highlighting therapeutic potential.

## Introduction

HIV remains one of the most significant public health challenges globally, affecting nearly 40 million people worldwide. In its later stages, people living with HIV-1 (PLWH) frequently experience a spectrum of neurological and neurocognitive complications, collectively referred to as HIV-associated neurocognitive disorders (HAND) [1–5]. These disorders are often exacerbated by co-occurring infections or illicit drug use, which further contribute to pronounced neurocognitive impairments. Epidemiological evidence indicates that illicit drug abuse is a leading factor in HIV transmission in the United States [6–8].

HIV-Tat, the trans-activator of transcription, is a protein secreted by infected cells. Unlike other viral proteins, Tat persists in the cerebrospinal fluid (CSF) of virally suppressed PLWH [9] and remains biologically active. Elevated Tat levels are strongly associated with substance abuse, including opioid use disorder (OUD). The interplay between HIV infection and OUD has emerged as a major concern in the post-HAART era. While antiretroviral therapy has significantly extended lifespan, neurological complications, including HAND and secondary central nervous system (CNS) abnormalities, remain prominent clinical challenges [10–14].

Opioids represent one of the most widely abused classes of psychoactive substances, with heroin being the most misused. Heroin is rapidly metabolized to morphine in the brain, making morphine the focus of most experimental studies. Among opioid receptor subtypes, the μ-opioid receptor (MOR) is the primary binding target for abused opioids such as morphine. Morphine profoundly alters immune function by modulating key cell populations, including phagocytes, T cells, and dendritic cells [15]. Opioids such as morphine have been shown to enhance HIV replication, disrupt blood–brain barrier integrity, impair innate and adaptive immune responses, and potentiate HIV protein-mediated neuroinflammation and oxidative stress [16, 17]. In parallel, HIV viral proteins, including Tat and gp120, together with opioids, exacerbate microglial activation, mitochondrial dysfunction, and neuronal injury, thereby accelerating HIV-associated neurodegenerative processes [5, 7, 18]. Neuroanatomical regions such as the striatum and hippocampus, which express high MOR densities, correlate with elevated viral titers in PLWH [19, 20]. Beyond direct modulation of neural responses to HIV, opioids also influence endogenous opioid peptide systems, further impacting CNS function. Despite advances in understanding HIV neuropathology and OUD, the precise mechanisms by which morphine exacerbates HIV-associated neurodegeneration remain poorly understood. Elucidating these molecular interactions is critical for identifying potential therapeutic targets for HAND.

Maintenance of genomic integrity is essential for neuronal survival and function. Neurons depend on tightly regulated DNA damage response (DDR) pathways to repair lesions induced by metabolic stress and exogenous insults. Failure or inaccuracy in DNA repair leads to genomic instability, triggering signaling cascades that contribute to neuronal dysfunction, neurodegeneration, and apoptosis [21, 22]. Consistent with this, mutations in DNA repair genes are strongly associated with several neurodegenerative disorders [23, 24]. Among the different forms of DNA damage, DNA double-strand breaks (DSBs) are particularly deleterious because they pose a major threat to neuronal survival and function.

The DDR is a highly coordinated signaling network composed of sensors, mediators, transducers, and effectors that collectively preserve genomic fidelity. Central to this network are the phosphatidylinositol 3-kinase–related kinases ataxia telangiectasia and Rad3-related (ATR), ataxia telangiectasia mutated (ATM), and DNA-dependent protein kinase (DNA-PK). ATR primarily responds to replication stress and UV-induced DNA lesions, whereas ATM and DNA-PK coordinate DSB repair through homologous recombination (HR) and nonhomologous end joining (NHEJ), respectively [25–27].

Among the excision repair cross-complementation (ERCC) family proteins, ERCC2 plays a critical role in maintaining genomic integrity and functions in the nucleotide excision repair (NER) pathway. Together with ERCC1-4, ERCC2 contributes to the repair of bulky DNA adducts, interstrand cross-links, and DNA DSBs through structure-specific repair mechanisms [28]. In addition, ERCC6 is a DNA-binding protein essential for transcription-coupled NER and other DNA repair processes. ERCC6 possesses ATPase activity and interacts with transcriptional and DNA repair factors to facilitate the assembly of repair complexes at sites of DNA damage [29].

DDR efficiency is tightly linked to chromatin organization and epigenetic regulation. Large Organized Chromatin Lysine domains (LOCKs) are expansive heterochromatic regions enriched in repressive histone modifications, contributing to higher-order nuclear architecture and transcriptional control [30]. Within LOCKs, histone H3 lysine 9 (H3K9) modifications are key regulators: H3K9 trimethylation (H3K9me3) marks heterochromatin and promotes genomic stability, whereas H3K9 acetylation (H3K9ac) marks transcriptionally permissive chromatin. The dynamic balance between these antagonistic modifications shapes global epigenetic landscapes and local DDR responses [31]. Herpes simplex virus 1 (HSV-1) induces replication fork collapse and DSBs, activating the ATM and ATR pathways, which are essential for viral replication [32] Similarly, RNA viruses such as Rift Valley fever virus manipulate DDR markers to facilitate infection [33, 34] Conversely, DDR components contribute to antiviral defense; DNA-PK acts as a cytoplasmic DNA sensor, while etoposide-induced DNA damage activates stimulator of interferon genes (STING), ATM, and NF-κB to stimulate interferon production [33–35]. To date, the effects of Tat protein and morphine co-exposure on DDR-driven innate immunity, particularly regarding immune cell recruitment and functional responses, remain largely unexplored.

This study aimed to investigate how HIV-Tat protein and morphine, individually and in combination, influence DDR and double-strand break (DSB) accumulation in the brain with or without dimethyl fumarate (DMF) treatment. DMF is an anti-inflammatory and immunomodulatory compound that activates the Nrf2 antioxidant pathway, enhancing cellular defense against oxidative stress [36–38]. It is clinically approved for psoriasis and multiple sclerosis, where it reduces relapse rates, slows disease progression, and decreases neuroinflammation [39]. DMF has also been proposed as an adjunct to combination antiretroviral therapy (cART) to mitigate systemic and CNS complications of HIV infection [36, 38], although its specific effects on microglial function in HIV-associated neuroinflammation remain unclear. In this context, we examined the cerebral cortex of Tat transgenic (iTat-Tg) mice to determine whether morphine exacerbates Tat-induced genomic instability and associated neuropathological alterations, and whether DMF can mitigate these effects. We further examined how alterations in LOCK-associated chromatin states influence ATM activation and the phosphorylated form of variant histone H2AX (γH2AX) formation both in vitro and in vivo. We hypothesized that HIV-Tat protein and morphine synergistically disrupt LOCK integrity, causing loss of H3K9me3-enriched heterochromatin and gain of H3K9Ac-associated open chromatin [40]. This epigenetic imbalance weakens DDR signaling by impairing ATM activation and γH2AX propagation, promoting DSB accumulation in microglia. The resulting genomic instability amplifies neuroinflammatory responses by activating innate immune pathways, contributing to neuropathological changes associated with HAND.

## Materials and methods

### In vitro studies

#### HIV‑1 Tat protein

The HIV-Tat protein was obtained from the National Institutes of Health AIDS Research and Reference Reagent Program (Cat # 2222). The recombinant Tat protein had a purity level of > 95%.

### Human microglia

**Fig. 1:**
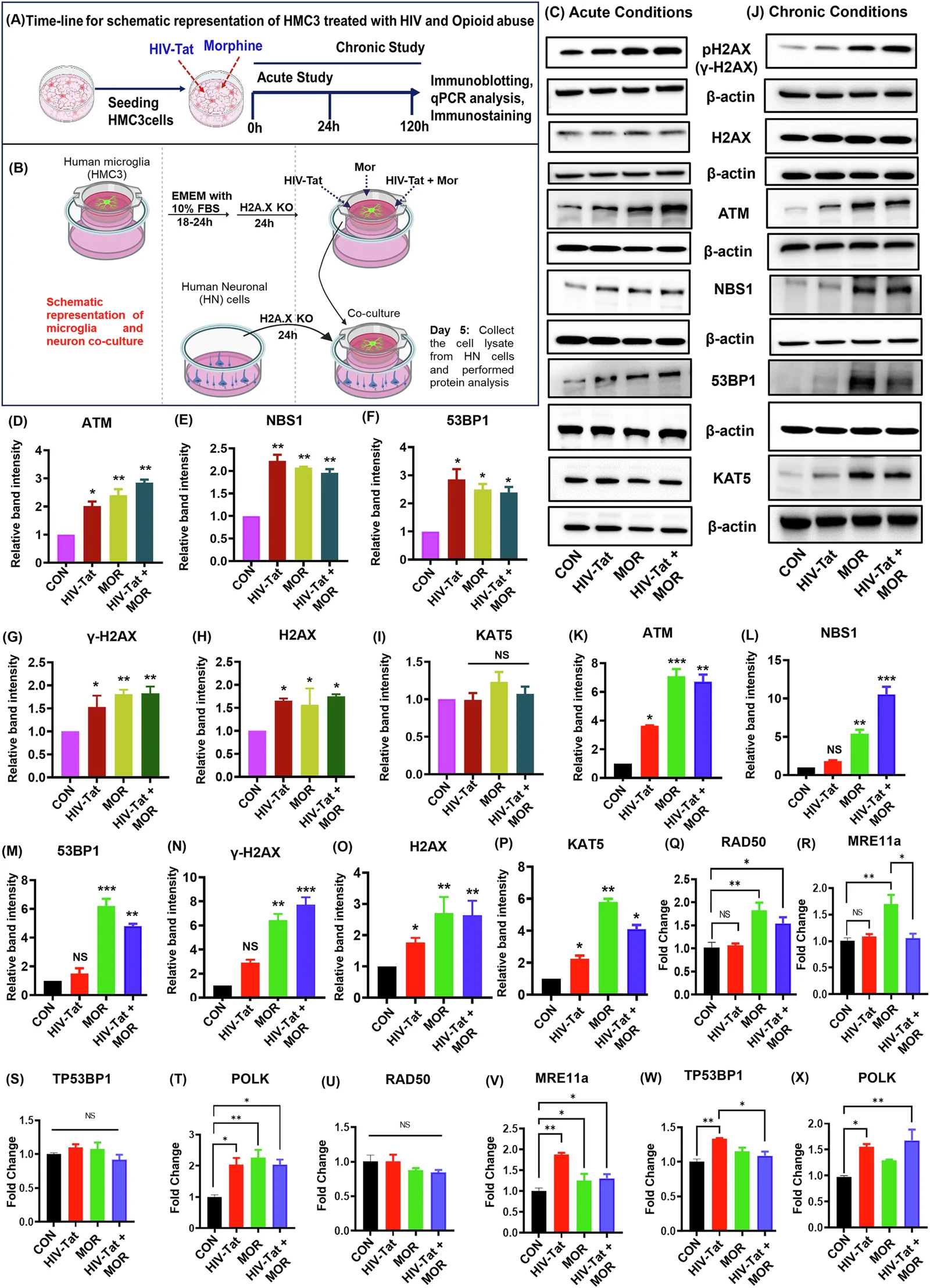
Induction of ATM activation and H2AX phosphorylation in human microglial cells (HMC3). **A** Timeline for schematic representation of HMC3 treated with HIV-Tat (50 ng/mL) and morphine (10 µM) for 24 h and 5 days. **B** Schematic representation of microglia-neuron co-culture. **C** Western blot analysis of DDR (DSBs) and KAT5/TIP60 expression in microglial cells exposed to HIV-Tat protein and morphine for 24 h. **D**–**I** Quantification of (**D**) ATM, **E** NBS1, **F** 53BP1, **G** phospho-H2AX, **H** H2AX, and **I** KAT5 (TIP60) from (**C**). **J** Analysis of DDR (DSBs) and TIP60 expression in microglial cells treated with HIV-Tat and morphine for 5 days. **K**–**P** Quantification of (**K**) ATM, **L** NBS1, **M** 53BP1, **N** phospho-H2AX, **O** H2AX, and **P** KAT5 (TIP60) from (**J**), with protein levels normalized to β-actin. (**O**–**R** and **S**–**V**) mRNA expression levels of DNA repair proteins MRE11, RAD50, TP53BP1, and POLK in human microglia under both acute (**Q**–**T**) and chronic conditions (**U**–**X**), analyzed by RT-qPCR using the 2^–ΔΔCT^ comparative method. The observed alterations in expression levels of DNA repair proteins occurred in human microglia treated with HIV-Tat protein and morphine compared to control groups. Data are presented as mean ± SEM; *n* = 3 per group. ^*^P < 0.05, ^**^P < 0.01, ^***^P < 0.001, and NS = non-significant by one-way ANOVA followed by Tukey’s post-hoc multiple comparison test.

Human Microglia (HMC3) cells were procured from ATCC (#CRL-3304, USA) and maintained in 10% EMEM (catalog# 30-2003, ATCC, USA) media supplemented with 5 mL of Penicillin/Streptomycin (P/S, Cat. No. 0503) to achieve a final concentration of 1% antibiotic/antimycotic solution^1^. To model acute and chronic exposure paradigms, HMC3 cells were treated with HIV-Tat protein (50 ng/mL) either alone or in combination with morphine (10 μM). Acute exposure was conducted for 24 h, whereas chronic exposure was maintained for 120 h (~5 days) under standard culture conditions (37 °C, 5% CO₂). For the chronic treatment paradigm, culture media were replaced every 40–42 h with freshly prepared complete media supplemented with the respective concentrations of Tat and morphine to ensure sustained exposure throughout the experimental period, while saline-treated HMC3 cells were used as the control group. Following completion of the treatment period, cells were harvested for downstream analyses. A schematic representation of the experimental design is shown in Fig. 1A, and all experiments were performed in triplicate (*n* = 3).

### siRNA transfection studies

H2A.X siRNA (Assay ID: s226270) was purchased from Invitrogen, and transfections were performed using Lipofectamine 3000 following the manufacturer’s protocol for 6-well plates. For each transfection, 150 μL of Opti-MEM was added to an Eppendorf tube, followed by the indicated concentrations of H2A.X siRNA (30 pMol, 3 nMol, or 30 nMol) or an equal amount of scrambled siRNA. In a separate tube, 9 μL of Lipofectamine 3000 was diluted in 150 μL of Opti-MEM. After briefly vortexing each solution, the siRNA and Lipofectamine mixtures were combined, gently mixed, and incubated at room temperature for 15 min. The resulting 250 μL siRNA–lipid complex was then added dropwise to HMC3 cells in 6-well plates, gently mixed, and returned to the incubator.

Knockdown efficiency was assessed by western blotting (Fig. 3A). Of the concentrations tested, 30 pMol siRNA produced approximately 70% reduction in H2A.X protein expression and was therefore used for all subsequent experiments. Following transfection, cells were incubated for 24 h before treatment with HIV-Tat (50 ng/mL) and/or morphine (10 μM) for 5 days. Immunoblotting was performed to verify target knockdown and evaluate DDR and inflammatory markers.

### Microglia–neuron co-culture

The human neuronal (HN) cells were purchased from ScienCell (Cat. #1520) and maintained in Neuronal Medium (NM, Cat. #1521), consisting of 500 mL basal medium, 5 mL Neuronal Growth Supplement, and 5 mL penicillin/streptomycin, in a humidified 37 °C incubator with 5% CO₂. Microglia–neuron co-culture workflow is illustrated in Fig. 1B. Human microglial HMC3 cells were seeded on polycarbonate membrane inserts (3 μm pore size; Thermo Scientific™ Nunc™ Transwell) at a density of 0.25 × 10⁶ cells/insert in EMEM supplemented with 10% fetal bovine serum (FBS). In parallel, HN cells were plated in 6-well plates at 0.25 × 10⁶ cells/well using DMEM containing 10% FBS. After an initial 18 h incubation to allow cell attachment, both HMC3 and HN cultures were transfected with H2A.X-targeting siRNA (30 pmol) following the manufacturer’s protocol. The knockdown period was maintained for 24 h. After knockdown, HMC3 cells on inserts were treated under the following conditions in EMEM containing 2% FBS: Control, HIV-Tat protein (50 ng/mL), morphine (10 μM), or HIV-Tat + morphine co-treatment. Recombinant HIV-Tat protein and morphine concentrations were selected based on previously established neuroHIV and opioid co-exposure models [41, 42]. At the same time, HN cells in the lower chamber were refreshed with neuronal maintenance medium (NM). The treated HMC3 inserts were then transferred above HN cultures to establish an indirect Transwell co-culture, enabling soluble factor exchange without direct cell–cell contact. The co-culture was maintained for 5 days.

### MTT cell viability assay

Microglia conditioned media (MCM) were initially collected following exposure of HMC3 to HIV-Tat with or without Morphine. HN cells were subjected to the following MCM with the following treatments [1] CON-Saline MCM [2], HIV-Tat MCM [3], Mor-MCM [4], HIV-Tat + Mor-MCM for 24 h. At the end of the treatment period, cells were subjected to an MTT cell viability assay [43, 44]. Briefly, HN cells were plated at approximately 1 × 10⁴ cells per well in 96-well plates and were incubated for 24 h at 37 °C. After incubation, the spent medium was aspirated, and 100 µL of MCM from different treatments was added to each well before incubating the plates for an additional 24 h. A working MTT solution was prepared by diluting the 100× MTT stock (50–100 mg/mL) in culture medium to obtain a final concentration of 0.5 mg/mL, and 100 µL of this solution was added to each well. The plates were then incubated for 4 h in a humidified 37 °C incubator with 5% CO₂. Following this incubation period, the MTT solution was carefully aspirated from each well, and the resulting formazan crystals were dissolved by adding 100 µL of DMSO. Absorbance was measured at 570 nm using a plate reader, with background correction performed using blank wells containing no cells and comparison made to untreated control wells.

### In vivo studies

#### Animals

The iTat-Tg mouse line expresses a “tetracycline-on (TET-ON)” system integrated into the astrocyte-specific glial fibrillary acidic protein (GFAP) promoter and coupled to the Tat_1–86_ coding gene, allowing astrocyte-specific Tat_1–86_ expression upon DOX administration [45]. This model enables focused studies on Tat-mediated dysregulation of neuronal and glial processes. In contrast, G-Tg (Tat null) mice harbor the TET-ON system but lack the Tat_1–86_ coding gene, rendering them incapable of Tat expression (Tat-null), but are highly suitable as control subjects [46]. Both iTat-Tg and G-Tg mice were derived from progenitors initially provided by Dr. Johnny J. He (Rosalind Franklin University of Medicine and Science, Chicago, IL) [45].

All experimental mice were male, aged 8–14 weeks, and housed 4–5 per cage in a temperature-controlled- (21 ± 2 °C) and humidity-controlled (50 ± 10%) vivarium under a 12:12 h light/dark cycle (lights on at 07:00 h) with ad libitum access to food and water. Male mice were selected to minimize variability in reward responses due to the estrous cycle. C57BL/6J mice were obtained from Jackson Laboratory (Bar Harbor, Maine, USA) and maintained in accordance with the NIH Guide for the Care and Use of Laboratory Animals in AAALAC-accredited facilities. All experimental protocols were approved by the Institutional Animal Care and Use Committee (IACUC) at the University of Florida, Gainesville, FL (protocols 202209933, approved April 5, 2022, and 202300000185, approved March 20, 2023). Future studies will include female mice to evaluate sex-dependent effects and better capture biological variability.

### Drug administration

To assess the in vivo effects of Tat protein and morphine on neuronal and glial function, male iTat-tg and G-tg mice were administered doxycycline hyclate (DOX; 100 mg/kg/day, Sigma-Aldrich, St. Louis, MO) or saline (10 μL/g body weight) via intraperitoneal (i.p.) injection for 7 consecutive days (*n* = 10–19). The Dox dose was selected based on previous studies demonstrating efficient Tat induction and biologically active protein levels during behavioral testing [47]. One group of iTat mice received a single dose of morphine sulfate (MOR; Sigma-Aldrich, St. Louis, MO) at 10 mg/kg body weight on Day 7, while another group was treated as follows: on Day 4 after initiation of DOX or saline, additional mice received either saline or escalating doses of morphine sulfate (MOR) twice daily at doses from 10 to 75 mg/kg as reported previously until day 7 [48]. The morphine dose was chosen based on prior studies showing robust behavioral and neurochemical effects without excessive toxicity, yet producing opioid physical dependence [48].

### DMF treatment groups

To evaluate potential therapeutic effects, additional groups of mice received DMF (30 mg/kg/day, p.o.) 20 min prior to morning DOX and/or morphine treatment for 7 days, following the regimen described by Garcia-Mesa et al. [49]. DMF was dissolved in 5% DMSO/50% Solutol/90% saline for administration. DMF was selected due to its well-established capacity to activate the Nrf2 antioxidant pathway, enhance endogenous cellular defense mechanisms, and reduce oxidative stress and neuroinflammation. These properties make it a promising candidate to counteract HIV-Tat- and morphine-induced oxidative DNA damage, chromatin remodeling deficits, and dysregulated inflammatory signaling. Administration of DMF in these groups enabled investigation of its efficacy in normalizing HIV-Tat/morphine-induced perturbations in neuronal and glial function, DDR pathways, and innate immune activation.

### Tissue preparation and immunohistochemistry

Mice were transcardially perfused with either sterile saline (0.9%) for molecular studies or 4% paraformaldehyde (PFA) for immunohistochemistry studies, and brains were removed. Brains for molecular studies were then snap-frozen in liquid nitrogen. Tissues for immunohistochemistry were additionally post-fixed in 4% PFA in phosphate-buffered saline (PBS, pH 7.4) for 24 h at 4 °C. Fixed tissues were washed thoroughly in PBS to remove residual fixative, dehydrated through a graded ethanol series (70%, 80%, 90%, 95%, and 100%; 1 h each), cleared in xylene (2 × 30 min), and embedded in paraffin wax at 60 °C. Paraffin blocks were sectioned at 5 µm thickness using a microtome and mounted on poly-L-lysine–coated glass slides. Sections were deparaffinized in xylene (2 × 10 min), rehydrated through a descending ethanol series (100%, 95%, 80%, 70%), and rinsed in PBS. Heat-induced antigen retrieval was performed in citrate buffer (10 mM sodium citrate, pH 6.0) at 95 °C for 15 min. Sections were then blocked with 5% normal donkey serum in PBS containing 0.3% Triton X-100 for 1 h at room temperature.

Primary antibodies against γH2AX, Iba-1, and H3K27Ac (1:500 in blocking buffer) were applied overnight at 4 °C. After PBS washes, sections were incubated with Alexa Fluor–conjugated secondary antibodies for 1 h at room temperature. Nuclei were counterstained with Hoechst, and sections were mounted using antifade medium. Fluorescence images were acquired using an FV3000 confocal microscope.

### Postmortem human brain tissues

Frontal cortical tissue samples from uninfected controls and HIV-positive individuals were obtained from the National NeuroAIDS Tissue Consortium (NNTC) and the National Neurological AIDS Bank (NNAB) at UCLA. HIV-positive participants were 30–60 years old, had confirmed HIV encephalitis, and had been on cART therapy for a minimum duration of 6 months; the cohort included three males and three females, all receiving continuous treatment. Clinical diagnosis followed DSM-5 criteria, including only cognitive impairment attributable to HIV and classified under neurocognitive disorders (NCDs). Individuals with vascular disease, infection, incarceration, inflammatory or metabolic disorders, structural brain abnormalities, trauma, delirium, or other systemic/genetic conditions were excluded. Samples were further stratified by viral load and CD4 count (HIV ≥ 200, CD4NC ≤ 50).

### Western blot analysis

Protein lysates (20 μg) from mouse brain tissues, human microglia and neurons, and human brain samples were separated on 4–20% gradient SDS-PAGE gels and transferred to PVDF membranes. Membranes were blocked with EveryBlot blocking buffer (#12010020, Bio-Rad) for 10 min and incubated overnight at 4 °C with primary antibodies against ATM, phospho-ATM (Ser1981), H2AX, phospho-H2AX (Ser139), ARID1A, BRG1, NBS1, 53BP1, ERCC1, ERCC2, ERCC3, ERCC6, ERCC8, AIM2, NLRP3, H3K27Ac, H3K9Ac, H3K27me3, H3K9me3, STING, cGAS, Caspase 1, and β-actin. On the following day, membranes were incubated with HRP-conjugated secondary antibodies for 1 h at room temperature. Protein signals were visualized using the Clarity Max Western ECL Substrate (1705062, Bio-Rad) and analyzed with ImageJ software (NIH). Band intensities were normalized to loading controls (β-actin) and expressed as fold change relative to controls as described by [50]. Antibody details are listed in Supplementary Table 1.

### Immunofluorescence analysis

Human microglia (1 × 10⁴ cells) were seeded on glass slide chambers and grown to 70–80% confluency before treatment with HIV-Tat protein (50 ng/mL) for 24 h. Cells were fixed in 4% PFA for 20 min, permeabilized with 0.1% Triton X-100 in PBS for 5 min, and blocked with 2% bovine serum albumin for 1 h at room temperature. Primary antibodies against BRG1 and H3K27Ac (1:500) were applied overnight at 4°C. Secondary antibodies conjugated with Alexa Fluor® 488 (green) or 555 (red) were applied at a 1:2000 dilution. Negative controls were processed without primary antibodies. Nuclei were stained with 0.2 μg/mL DAPI, and fluorescent signals were visualized using an FV3000 confocal microscope.

### RT-qPCR analysis

Microglial cells were homogenized, and total RNA was extracted using Trizol reagent. RNA (2 μg) was reverse transcribed into cDNA using the iScript™ Advanced cDNA Synthesis Kit (Bio-Rad) following the manufacturer’s instructions. RT-qPCR was performed with 1:20 diluted cDNA and SYBR Green master mix on a CFX96™ Real-Time System (Bio-Rad) under the following conditions: initial denaturation at 95°C for 20–30 s (1 cycle), denaturation at 95 °C for 1–3 s (40 cycles), and annealing/extension at 60 °C for 20 s (40 cycles), followed by a melt curve from 60 °C to 95 °C (1 cycle). Primers for RAD50, MRE11a, TP53BP1, and POLK were purchased from Bio-Rad. Relative gene expression was calculated using the 2^−ΔΔCt^ method, normalized to GAPDH, and expressed as fold change relative to controls [51].

### Behavioral studies—novel object recognition (NOR) test

Learning and memory were assessed using the NOR assay as previously described [47, 52]. The task consisted of three 10-min phases during which mice freely explored two objects (A and B). For each phase, the time spent attending to each object was recorded. Phases were separated by a 10-min inter-trial interval to support consolidation. During phases 1 and nd 2, objects were a pair of dice located in consistent locations to begin each exposure [47, 52]. In phase 3, object B was replaced with a novel object (a marble). Upon the completion of testing, observers blinded to the treatment measured the time mice spent attending to each object in each trial. Attending to an object was defined as the duration of time a mouse spent in physical contact with the object, using any body part other than the tail, or whenever it was within 0.5 cm of the object, facing it, and engaged in active exploration. In phases 1 and 2, both objects were identical dice placed in consistent locations. In phase 3, object B was replaced with a novel object (a marble). For each phase, the observers recorded the time spent attending to each object using stopwatches [47, 52]. Data are presented as a percentage recognition index (RI) for object B: RI = [(time attending to object B/time attending to object A + B)] × 100.

### Statistical analysis

Detailed information regarding sample size and the statistical tests used for each experiment is provided in the manuscript. For in vitro experiments, a sample size *n* = 3 per condition was used. Data represent three independent experiments. In the in vivo mouse studies, group sizes varied from *n* = 10–19 animals, which provided ≥80% power to detect biologically meaningful differences at a two-tailed α level of 0.05. All data are expressed as mean ± SEM. Student’s two-tailed paired *t*-tests were used for point-to-point comparisons as appropriate. One-way ANOVA followed by Tukey’s post hoc test was performed; otherwise, multiple group comparisons were performed using GraphPad Prism 10.0 (La Jolla, CA, USA). Comparisons between normal and HIV-positive human subjects were performed using a two-tailed unpaired *t*-test. A *P*-value ≤ 0.05 was considered statistically significant.

## Results

### In vitro analysis of DNA damage and repair in human microglial cells

#### HIV-Tat and morphine co-treatment induces DNA damage signaling in microglia

To investigate how HIV-Tat protein and opioid exposure affect DNA integrity, human microglial HMC3 cells were treated with recombinant HIV-Tat, morphine, or their combination. Western blot analysis revealed a time-dependent induction of DDR proteins across all treatment groups. Acute exposure (24 h) significantly increased phosphorylated H2AX (γH2AX, Ser139), total H2AX, phosphorylated ATM (pATM, Ser1981), total ATM, NBS1, 53BP1, and KAT5 (Fig. 1C–I). Co-treatment with HIV-Tat and morphine elicited a synergistic amplification of these markers, consistent with enhanced DSB signaling. Chronic exposure (5 days) further elevated DDR protein expression, with the greatest induction observed in the HIV-Tat + morphine combination group (Fig. 1J–P), suggesting sustained genotoxic stress and activation of DNA damage signaling pathways that may contribute to impaired genomic stability.

### Modulation of DNA repair genes by HIV-Tat protein and morphine

We examined the expression of downstream genes in the ATM pathway, including MRE11a, RAD50, TP53BP1, and POLK, which are key mediators of DNA repair. Real-time PCR analysis showed selective upregulation of RAD50 and MRE11a in morphine and HIV-Tat + morphine groups during acute exposure, while TP53BP1 remained unchanged. POLK was elevated across all groups (Fig. 1Q–T). Under chronic treatment, MRE11a, TP53BP1, and POLK remained upregulated, whereas RAD50 levels were largely unchanged (Fig. 1U–X), suggesting selective recruitment of repair pathways to counteract persistent DNA damage.

### Alterations in DNA repair protein expression

**Fig. 2:**
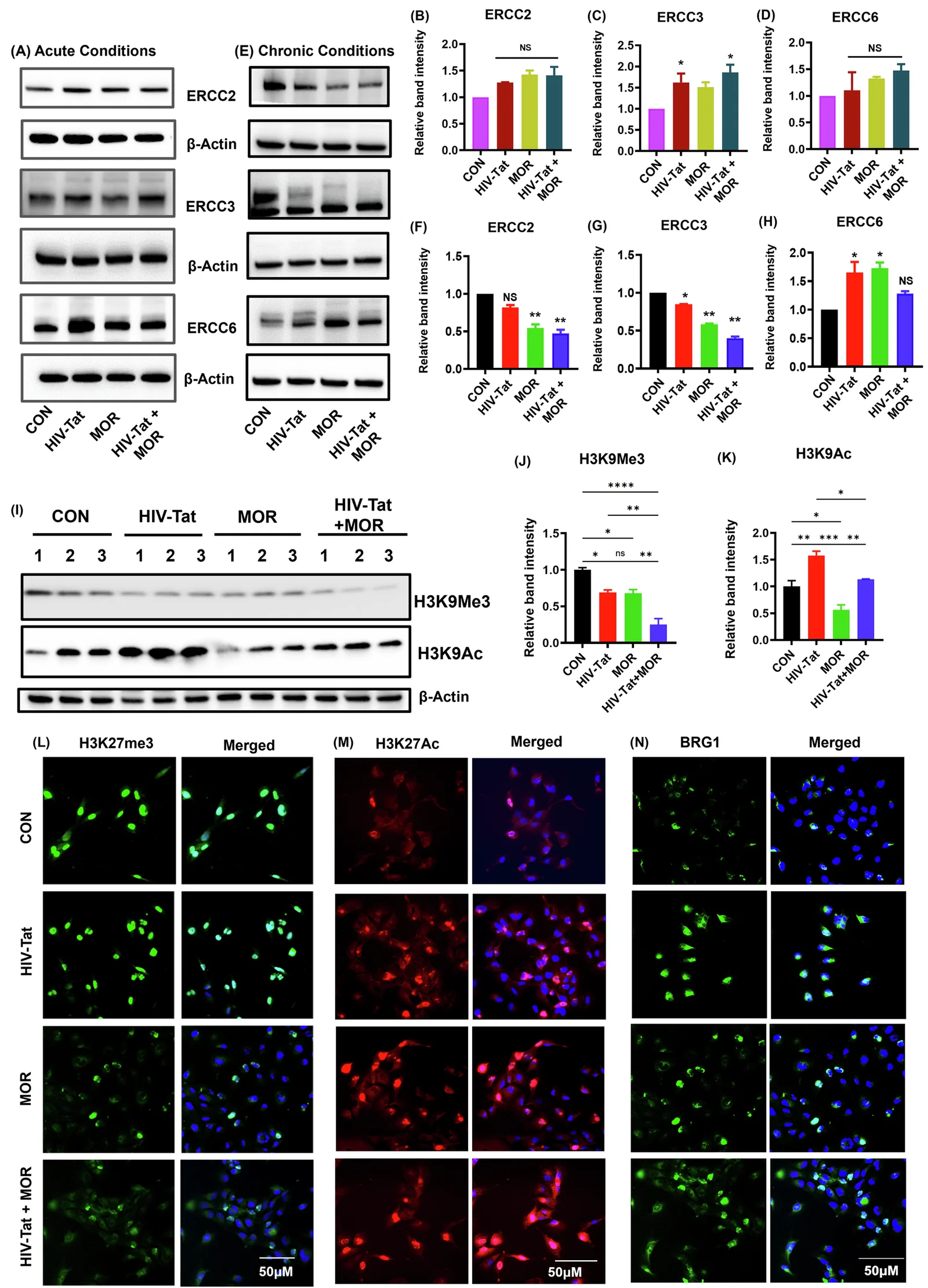
Alterations of DNA repair proteins including excision repair cross-complementing groups in HMC3. **A** Western blot analysis of DNA repair proteins in microglial cells exposed to HIV-Tat protein and morphine, alone or in combination, for 24 h. **B**–**D** Quantification of (**B**) ERCC2, **C** ERCC3, **D** ERCC6 shown in (**A**). **E** Reduction of DNA base excision repair proteins in microglial cells treated with HIV-Tat protein and morphine, alone or in combination, for 5 days. **F**–**H** Quantification of (**F**) ERCC2, **G** ERCC3, **H** ERCC6 shown in (**E**). **I** Western blot analysis of the LOCK system, H3K9Me3 and H3K9Ac, in human microglial cells exposed to HIV-Tat protein and morphine, alone or in combination, for 5 days. **J**, **K** Corresponding bar graphs with protein levels normalized to β-actin. **L**, **M** Immunofluorescence analysis of H3K27Me3 and H3K27Ac in human microglia exposed to HIV-Tat protein and morphine, alone or in combination, under chronic conditions; scale bar = 50 µm. **N** Immunostaining of BRG1 expression in human microglial (HMC3) cells treated for 5 days with HIV-Tat protein and morphine, alone or in combination: magnification 40×, scale bar = 50 µm. Data are presented as mean ± SEM; *n* = 3 per group. ^*^P < 0.05, ^**^P < 0.01, ^***^P < 0.001, and NS = non-significant by one-way ANOVA followed by Tukey’s post-hoc multiple comparison test.

We next examined expression of DNA repair proteins from the ERCC family, including ERCC2, ERCC3, and ERCC6, in HMC3 cells exposed to HIV-Tat, morphine, or their combination. Western blot analysis of HMC3 cells treated chronically (5 days) revealed a significant reduction in ERCC2 and ERCC3 levels, with concurrent upregulation of ERCC6 across all treatment groups relative to control (Fig. 2A–H). By contrast, no significant changes were observed under acute conditions. These findings indicate impaired NER capacity under prolonged exposure, with ERCC6 possibly reflecting compensatory activation of transcription-coupled repair pathways. Acute exposure did not alter these proteins, highlighting the temporal dynamics of DNA repair dysfunction.

### Chromatin remodeling and LOCK disruption

Given the interplay between DNA repair and chromatin organization, we examined Large Organized Chromatin K9-modified domains (LOCKs). Western blot analyses demonstrated a pronounced loss of repressive mark H3K9me3, accompanied by a corresponding increase in activating marks H3K9ac (Fig. 2I–K), under chronic conditions. Aligning with these observations, immunostaining revealed a marked decrease in H3K27me3 and a reciprocal elevation in H3K27ac following HIV-Tat or morphine exposure, alone or in combination, under chronic treatment conditions (Fig. 2L–M). Furthermore, we showed elevated BRG1 expression, particularly in HIV-Tat and HIV-Tat + morphine co-treated cells (Fig. 2N), indicating recruitment of SWI/SNF chromatin remodelers and disruption of LOCK integrity.

### H2A.X knockdown attenuates DDR and inflammatory signaling

**Fig. 3:**
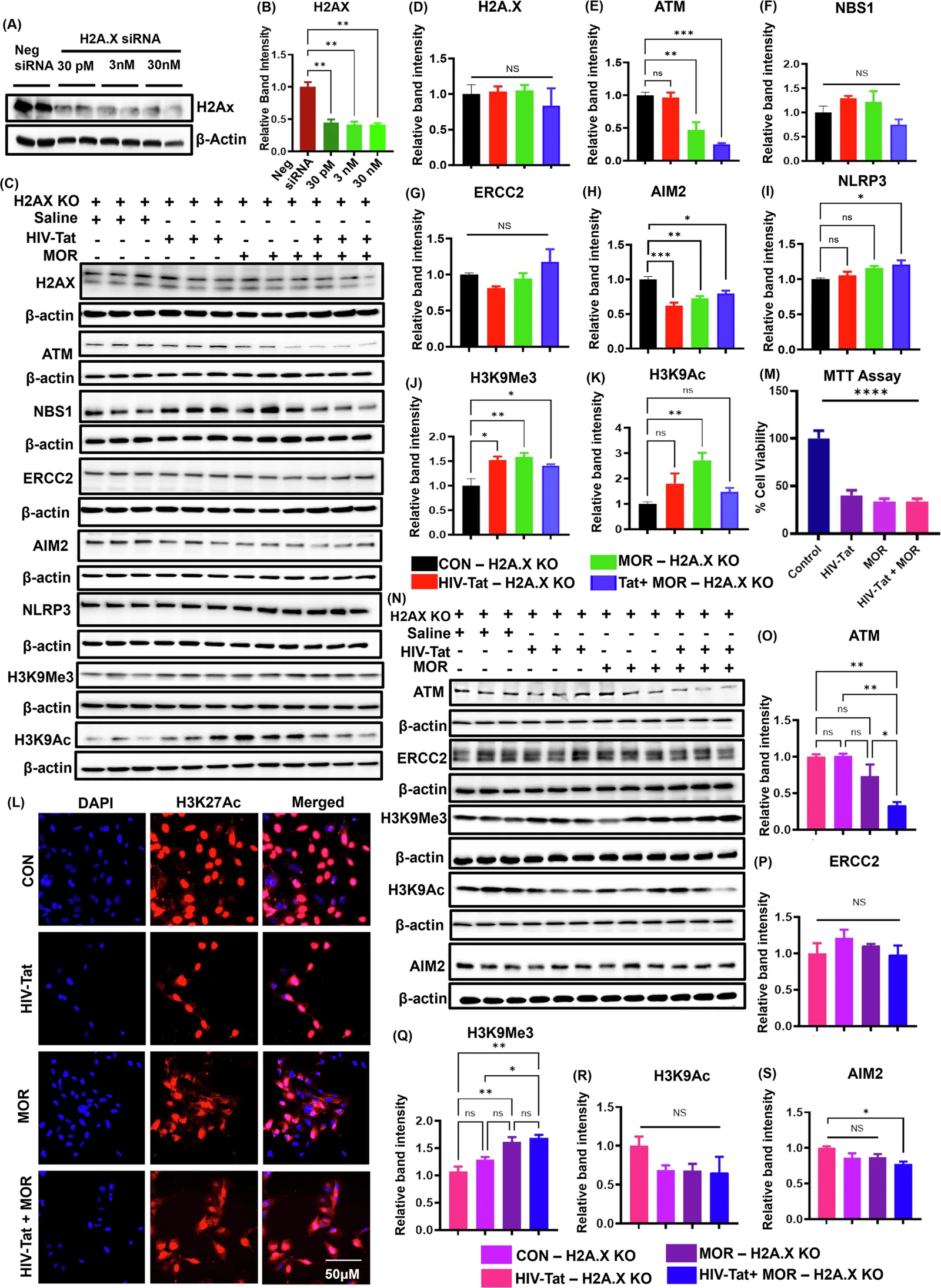
H2A.X knockdown alters DDRs, inflammasome activation, and chromatin states in neuron–microglia co-cultures exposed to HIV-Tat protein and morphine. **A**, **B** Validation of H2A.X knockdown in human microglia treated with H2A.X siRNA (30 pM, 3 nM, 30 nM). Western blot (**A**) and quantification (**B**) confirm dose-dependent reduction of H2A.X relative to negative siRNA control. **C**–**K** Western blot analysis of DSB and DNA repair pathways in H2A.X-deficient microglia exposed to HIV-Tat protein and morphine for 24 h. **C** Representative blots for H2A.X, ATM, NBS1, ERCC2, AIM2, NLRP3, H3K9me3, and H3K9Ac. Quantification of (**D**) H2A.X, **E** ATM, **F** NBS1, **G** ERCC2, **H** AIM2, and **I** NLRP3, and protein levels normalized to β-actin. **J**, **K** Quantification of chromatin modifications, including repressive H3K9me3 and permissive H3K9Ac marks, in H2A.X-depleted microglia under inflammatory challenge. **L** Immunofluorescence imaging of H3K27Ac in HMC3-H2A.X KO cells exposed to saline control, HIV-Tat protein, MOR, or HIV-Tat protein + MOR under chronic conditions (5 days). Representative images (DAPI: blue; H3K27Ac: red) show reduced acetylation following HIV-Tat protein and MOR exposure, consistent with Western blot results. Scale bar: 50 μm. **M** MTT assay showing neuronal viability in co-cultures. Data are presented as mean ± SEM; *n* = 6 per group. ^****^P < 0.0001 by one-way ANOVA followed by Tukey’s post-hoc multiple comparison test. **N**–**S** Western blot analyses of H2A.X knockdown HN cells co-cultured with activated HMC3 microglia and treated with saline, HIV-Tat protein, morphine (MOR), or HIV-Tat protein + MOR. **N** Representative blots for ATM, ERCC2, H3K9me3, H3K9Ac, and AIM2. Quantification of (**O**) ATM, **P** ERCC2, **Q** H3K9me3, **R** H3K9Ac, and **S** AIM2, and protein levels normalized to β-actin. Data are presented as the mean ± SEM; *n* = 3 per group. ^*^P < 0.05, ^**^P < 0.01, ^***^P < 0.001, and NS = non-significant by one-way ANOVA with Tukey’s post hoc test.

To assess H2A.X’s role in DDR, HMC3 cells underwent siRNA-mediated H2A.X knockdown for 24 h, followed by exposure to HIV-Tat, morphine alone, or in combination under chronic treatment paradigms. Loss of H2A.X significantly reduced ATM and NBS1 expression, highlighting its role in sustaining canonical DDR pathways (Fig. 3C–F). Knockdown also attenuated AIM2 inflammasome activation and partially restored ERCC2 levels (Fig. 3C, G–I). Repressive chromatin marks, including H3K9me3, increased, whereas H3K9Ac remained unchanged, further supporting H2A.X’s role in coordinating DNA damage and chromatin responses (Fig. 3C, J–K). Moreover, immunostaining analysis showed that H3K27ac levels remained unchanged following H2A.X knockdown in HMC3 cells, regardless of treatment condition under chronic exposure (Fig. 3L).

### Interplay of chromatin regulation and inflammatory signaling in neuron-glia co-culture model

To assess the impact of HIV-Tat protein and morphine-induced microglial activation on neuronal viability, HMC3 cells were treated with HIV-Tat, morphine alone or in combination for 5 days. Microglia-conditioned media (MCM) from these treatments were applied to HN cultures for 24 h, followed by assessment of neuronal viability using the MTT assay. MCM from HIV-Tat-treated microglia, particularly in combination with morphine, significantly reduced neuronal survival (Fig. 3M). In co-culture experiments, neuronal alterations were associated with converging pathways, including repressive chromatin remodeling (LOCK system) and inflammatory signaling during neuron-glia interactions. Western blot data revealed that siRNA-mediated H2A.X knockdown in neurons increased H3K9me3, decreased H3K9Ac, and reduced ATM expression, while ERCC2 remained unchanged, accompanied by marked downregulation of AIM2 (Fig. 3N–S), highlighting the role of DDR in neuron-glia interactions.

### In vivo validation in iTat transgenic mice

#### HIV-Tat protein and morphine co-treatment induces microglial activation and DNA damage

**Fig. 4:**
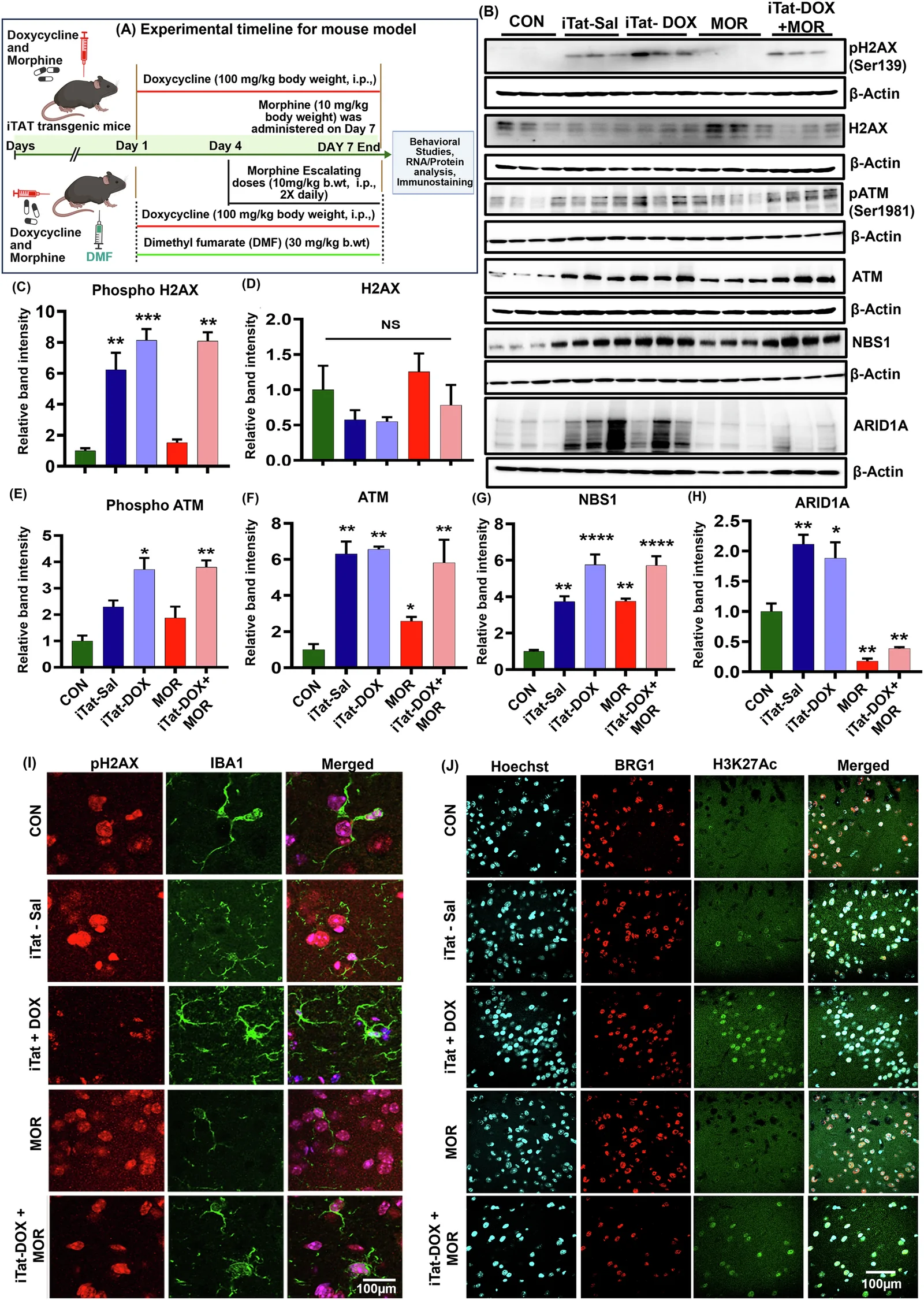
Induction of ATM activation and H2AX phosphorylation in DOX-induced iTat mice treated with morphine alone or in combination. **A** Experimental timeline for the iTat transgenic mouse model. **B** Brain cortical tissues were collected from DOX-induced iTat mice and from mice treated with morphine alone or in combination for Western blot analysis. Corresponding quantifications are shown in (**C**–**H**). Quantification of **C** phospho-H2AX (Ser139), **D** total H2AX, **E** phospho-ATM (Ser1981), **F** total ATM, **G** NBS1, and **H** ARID1A, normalized to β-actin. Control samples and morphine-treated tissues from Null mice, as well as DOX and DOX + morphine treatments in inducible iTat mice, were included. Comparisons were made between control vs iTat saline, control vs morphine, iTat saline vs iTat DOX, and iTat DOX vs iTat DOX + morphine, and morphine vs iTat DOX + morphine. Data are presented as mean ± SEM; *n* = 3/group. ^*^P < 0.05, ^**^P < 0.01, NS = non-significant by one-way ANOVA with Tukey’s post hoc test. **I** Immunofluorescence staining of nuclear γH2AX in the cortex of iTat mice treated with DOX with or without morphine, showing γH2AX induction and co-localization with the microglial marker Iba-1. Scale bar: 100 µm. **J** Representative immunostaining images showing H3K27Ac and BRG1 expression in the cortex of HIV-iTat mice following DOX induction with or without morphine. Scale bar: 50 µm.

To extend our in vitro findings, we used inducible Tat (iTat) transgenic mice, in which Tat expression is restricted to astrocytes upon DOX administration. At the end of the experiment, iTat transgenic mice received a single dose of morphine to model acute exposure. Western blot analysis showed significant induction of γH2AX (Ser139) and phosphorylated ATM (Ser1981) in DOX-induced iTat mice compared with non-transgenic or morphine-alone mice (Fig. 4B–F). Co-exposure to DOX and morphine further amplified these DNA damage markers, indicating a synergistic effect. Elevated NBS1, along with modulation of ARID1A and BRG1, reflected impaired MRN complex function and compensatory chromatin remodeling under sustained genotoxic stress (Fig. 4G, H, J). ARID1A levels were downregulated in the brains of mice induced with DOX + morphine. Immunofluorescence staining confirmed colocalization of H2A.X with Iba-1, a microglial marker, supporting our observations (Fig. 4I). These results indicate that H2A.X is predominantly associated with microglia.

### ERCC-mediated DNA repair and chromatin remodeling in mouse brain

**Fig. 5:**
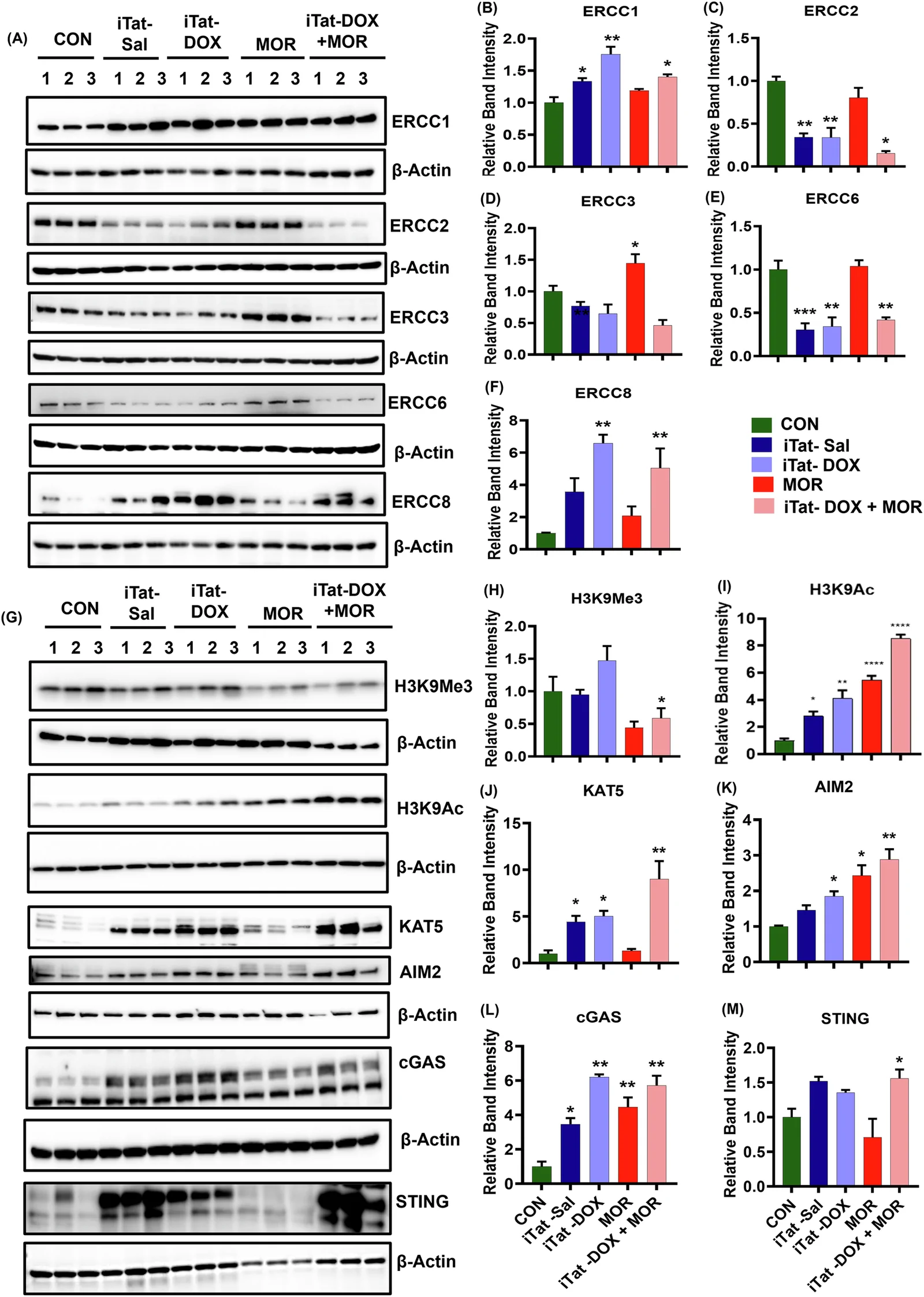
Dysregulation of DNA repair proteins and LOCK protein expression in DOX- and morphine-induced iTat mice. **A** Western blot analysis of DNA repair proteins, including excision repair cross-complementing groups (ERCC1–3, 6, and 8), from cortical tissues of transgenic mice. **B**–**F** Quantification of ERCC1 (**B**), ERCC2 (**C**), ERCC3 (**D**), ERCC6 (**E**), and ERCC8 (**F**) shown in (**A**). **G** Western blot analysis of LOCK system markers, H3K9Me3 and H3K9Ac, in the brain cortex of HIV-iTat transgenic mice following DOX induction with or without morphine treatment, with corresponding bar graphs (**H**, **I**), and protein levels normalized to β-actin. **G**, **J**–**M** Western blot analysis showing the expression of KAT5, AIM2 inflammasome components, cGAS, and STING in brain cortical tissues from DOX-induced and/or morphine-treated iTat transgenic mice. Representative blots are shown along with corresponding densitometric quantification in the accompanying bar graphs. Quantification of KAT5 (**J**), AIM2 (**K**), cGAS (**L**), and STING (**M**) protein levels normalized to β-actin. Comparisons were made between all experimental groups. Data are presented as the mean ± SEM; *n* = 3 per group. ^*^P < 0.05, ^**^P < 0.01, ^***^P < 0.001, and NS = non-significant by one-way ANOVA with Tukey’s post hoc test.

NER proteins were differentially regulated in iTat mice. Western blot analysis showed ERCC1 and ERCC8 were upregulated across DOX-induced and DOX + morphine-treated groups, indicating some level of compensatory repair activation. Conversely, ERCC2, ERCC3, and ERCC6 were selectively downregulated in DOX + morphine co-treated mice (Fig. 5A–F), consistent with impaired repair fidelity and chronic DNA damage accumulation. These alterations mirrored in vitro findings and highlighted the combinatorial impact of Tat and morphine on DNA repair machinery. Histone profiling further revealed a marked reduction in H3K9me3 accompanied by increased H3K9ac and H3K27ac in DOX-induced iTat mice, with morphine producing additional elevations in these acetylation marks (Fig. 5G–I). Correspondingly, KAT5/Tip60 and BRG1/ARID1A were upregulated, indicating a shift toward a more transcriptionally active chromatin state.

### Activation of cGAS-STING and AIM2 inflammasome pathways

Western blot analyses demonstrated that KAT5/Tip60, a lysine acetyltransferase, was markedly upregulated in total brain lysates from DOX-induced iTat mice, with further enhancement in DOX + morphine-treated mice brains (Fig. 5G, J, K). Increased KAT5/Tip60 expression promoted cGAS acetylation, thereby facilitating STING-dependent downstream proinflammatory signaling (Fig. 5G, L, M). Notably, activation of this pathway was accompanied by induction of the AIM2 inflammasome complex, indicating that Tip60-driven cGAS–STING signaling contributes to inflammasome activation in iTat mice exposed to DOX and morphine (Fig. 5G, K).

### DMF mitigates DNA damage and neuroinflammation

**Fig. 6:**
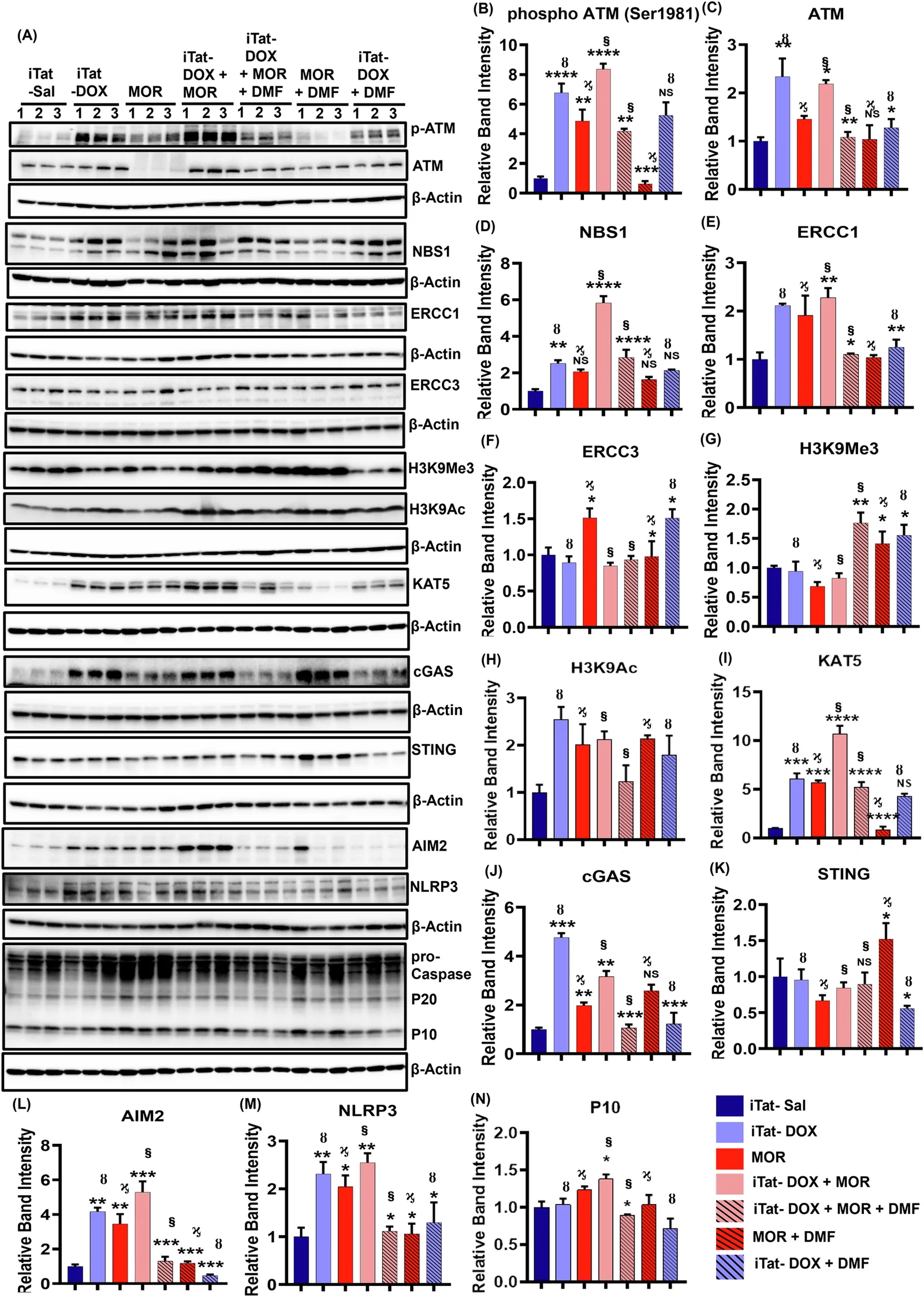
DMF attenuates DDR and inflammasome activation in the cortex of DOX- and morphine-induced iTat transgenic mice. **A** Western blot analysis showing the expression of phosphorylated ATM (p-ATM, Ser1981), total ATM, NBS1, ERCC1, ERCC3, cGAS–STING pathway components, and inflammasome proteins AIM2, NLRP3, and caspase-1 in brain cortical tissues from DOX- and morphine-treated iTat transgenic mice, with or without DMF administration. **B**–**J** Densitometric quantification of p-ATM (**B**), ATM (**C**), NBS1 (**D**), ERCC1 (**E**), and ERCC3 (**F**) reveals significant reductions following DMF treatment compared with DOX or morphine alone, while no changes were observed between other groups. **G**–**N** Western blot analysis of innate immune signaling components demonstrates that DMF suppresses cGAS–STING pathway activation and downstream inflammasome proteins AIM2 and NLRP3 in DOX- and morphine-treated iTat mice. Quantification of H3K9Me3 (**G**), H3K9Ac (**H**), KAT5 (**I**), cGAS (**J**), STING (**K**), AIM2 (**L**), NLRP3 (**M**), and caspase-1 (**N**) confirms reduced activation in DMF-treated groups (§DOX + Morphine + DMF vs DOX + Morphine, ϗMorphine + DMF vs Morphine, and ȣDOX + DMF vs DOX). Data are presented as mean ± SEM (*n* = 3 per group). ^*^P < 0.05, ^**^P < 0.01, ^***^P < 0.001, ^****^P < 0.0001, and NS = non-significant by one-way ANOVA with Tukey’s post hoc test.

DMF is a well-tolerated anti-inflammatory and immunomodulatory drug approved for the treatment of psoriasis and multiple sclerosis, where it has demonstrated neuroprotective and CNS-protective effects. Therefore, in the current study, we evaluated the therapeutic potential of DMF by administering it to DOX-induced iTat mice with or without morphine exposure. Mice received escalating morphine doses over 4 days to model acute-to-chronic opioid exposure. DMF treatment significantly attenuated phospho-ATM (ser1981), total ATM, and NBS1 expression, restoring these markers toward baseline levels observed in controls (Fig. 6A–D). ERCC1 expression was also reduced with DMF, whereas ERCC3 remained largely unchanged (Fig. 6A, E, F). Significantly, DMF enhanced H3K9me3-mediated transcriptional repression and stabilized LOCK domains, while modestly decreasing H3K9Ac (Fig. 6A, G, H). Moreover, DMF suppressed KAT5-cGAS-STING and AIM2/NLRP3 inflammasome signaling and Caspase-1 (P10) levels in DOX + Morphine induced mice, highlighting its capacity to dampen DNA damage-induced neuroinflammation and normalize microglial activity in the context of HIV-Tat and opioid co-exposure (Fig. 6A, I, N).

### Prophylactic DMF treatment ameliorates Tat protein- and/or morphine-induced deficits in NOR

**Fig. 7:**
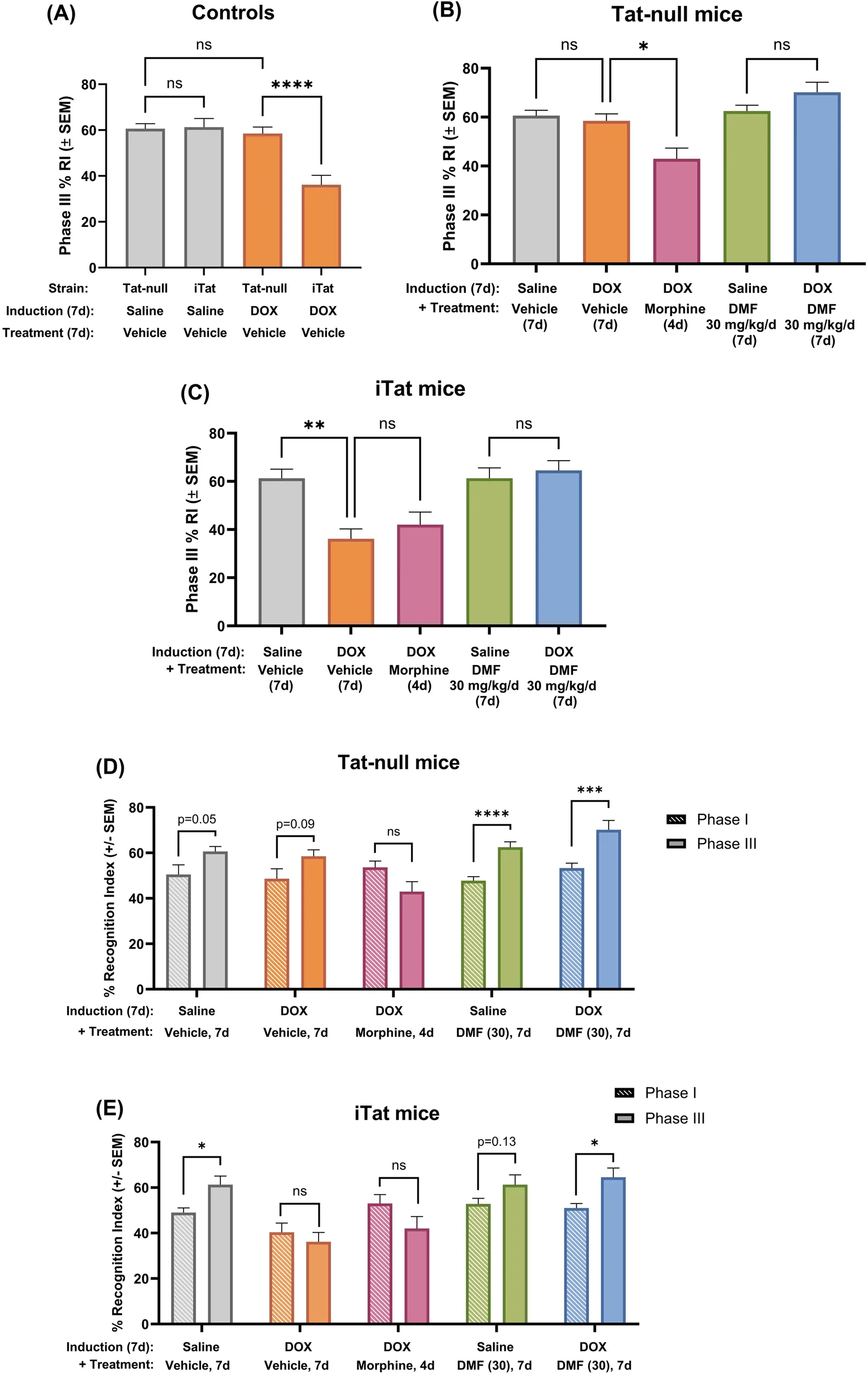
Tat protein-induced deficits in NOR are mitigated by prophylactic treatment with DMF. **A** Baseline and DOX Effects in Tat-null and iTat Controls. Phase III RI after 7 days of saline or DOX induction. Tat-null and iTat mice showed no difference in baseline performance. DOX impaired NOR only in iTat mice. *n* = 10–19 mice per bar. **B** Effects of Morphine and DMF on NOR performance in Tat-null Mice. Phase III RI in Tat-null mice after 7 days of saline or DOX induction, in addition to treatment with vehicle, morphine, or DMF (30 mg/kg, p.o.). DMF was administered 10 min prior to each saline or DOX induction. Morphine reduced NOR performance, while DOX and DMF alone did not. *n* = 11–23 mice per bar. **C** Effects of Tat Protein Induction, Morphine, and DMF in iTat Mice. Phase III RI in iTat mice after 7 days of saline or DOX induction, in addition to treatment with vehicle, morphine, or DMF (30 mg/kg, p.o.). DOX-mediated Tat expression and morphine impaired NOR performance without an observable additive effect. DMF alone had no effect but fully rescued the DOX-induced deficit. *n* = 10–20 mice per bar. **D** Phase I and Phase III Performance in Tat-null Mice. Phase I (exploration) and Phase III (recognition memory) RI in Tat-null mice under each treatment condition. **E** Phase I and Phase III Performance in iTat Mice. Phase I (exploration) and Phase III (recognition memory) RI in iTat mice under each treatment condition. DOX impaired Phase III performance, and DMF restored recognition memory without affecting Phase I exploration. Statistics: Phase I vs Phase III within each treatment group was analyzed by two-tailed paired *t*-tests. *n* = 10–20 mice per bar. ns = not significant; ^*^P < 0.05.

At baseline, saline-treated Tat-null and iTat mice showed no difference in performance in the NOR assay (Fig. 7A), validating that possession alone of the transgene does not affect recognition memory in the absence of DOX induction. As expected, DOX treatment had no effect on NOR performance in Tat-null mice, which lack the Tat gene, but produced significant impairment in iTat mice (Fig. 7A), consistent with expression of and exposure to HIV-Tat protein. In Tat-null mice, chronic morphine administration significantly reduced NOR performance (Fig. 7B), indicating that opioid exposure alone disrupts learning and memory. In contrast, in iTat mice, chronic morphine did not further exacerbate the DOX-induced NOR impairment, suggesting that the cognitive effects of morphine and Tat are not additive in this assay (Fig. 7C). Strikingly, repeated exposure to DMF at 30 mg/kg p.o. completely reversed the DOX-induced NOR deficit in iTat mice (Fig. 7C), restoring performance to control levels (Fig. 7C–E). Notably, DMF had no effect on NOR performance when administered alone in either genotype, indicating that it specifically mitigates Tat-dependent impairment of NOR without affecting baseline cognition.

### Validation in human HIV-positive brain tissues

#### γH2AX and ERCCs expression in frontal cortex

**Fig. 8:**
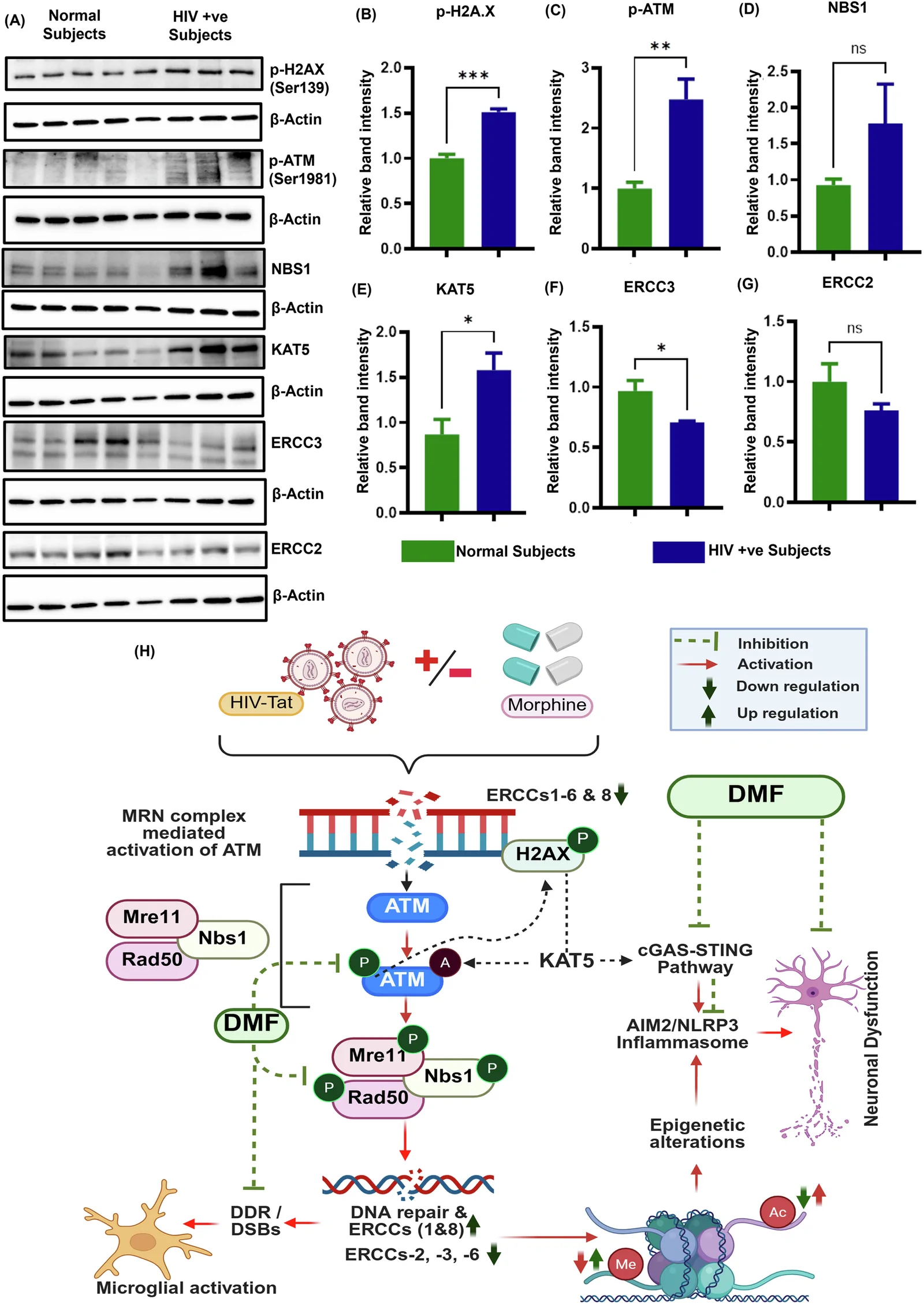
DNA damage markers and repair proteins in HIV+ subjects. **A** Western blot analysis showing upregulation of DNA damage markers in brain tissue from HIV+ subjects compared with normal controls (*n* = 4). **B**–**G** Quantification of phospho-H2AX (Ser139, **B**), phospho-ATM (Ser1981, **C**), NBS1 (**D**), KAT5 (**E**), ERCC3 (**F**), and ERCC2 (**G**) from the western blot in (**A**), with protein levels normalized to β-actin. Data are presented as mean ± SEM (*n* = 4 per group). ^*^P < 0.05, ^**^P < 0.01, ^***^P < 0.001, and NS = non-significant by two-tailed unpaired Student’s *t*-test. **H** Schematic illustrating how combined HIV-Tat protein expression and morphine exposure trigger KAT5/Tip60 activation and acetylation of chromatin remodeling complexes, leading to upregulation of the LOCK system and increased chromatin accessibility. This chromatin remodeling promotes DDR activation, characterized by phosphorylation of ATM and H2AX and recruitment of ERCC1 and NBS1 repair proteins. Persistent DNA damage facilitates cytosolic release of fragmented DNA, which activates the cGAS–STING pathway and downstream AIM2/NLRP3 inflammasomes, amplifying neuroinflammatory signaling in the brain cortex. Treatment with DMF attenuates KAT5/Tip60 acetylation, restores LOCK-associated heterochromatin integrity, and suppresses DDR and cGAS–STING/AIM2 inflammasome activation, thereby mitigating Tat protein- and morphine-induced neuroinflammation.

To validate our in vitro and in vivo results, we examined postmortem frontal cortex from HIV-positive individuals and matched controls (NNTC and NNAB). Western blot analysis showed significant increases in γH2AX, p-ATM, NBS1, and KAT5 in HIV-positive samples, indicating elevated DNA damage signaling (Fig. 8A–E). Conversely, ERCC2 and ERCC3 were markedly reduced, suggesting impaired NER (Fig. 8A, F, G). These reciprocal alterations mirrored findings in Tat- and morphine-treated HMC3 cells and iTat transgenic mice, highlighting the translational relevance of our models.

### Implications for neurodegeneration and microglial dysfunction

The observed accumulation of DNA DSBs and reduced levels of DNA repair proteins, including ERCCs, in HIV-positive human brains indicate persistent unrepaired DNA lesions, chromatin instability, and impaired DNA repair fidelity. These molecular deficits likely drive microglial activation, neuroinflammation, and neuronal dysfunction, contributing to HAND, consistent with our in vitro and in vivo findings.

Collectively, our results provide a hierarchical validation of the effects of HIV-Tat protein and opioid exposure on microglial DNA damage, impaired repair mechanisms, chromatin remodeling, and activation of innate immune pathways. Importantly, DMF treatment mitigates DNA damage, stabilizes chromatin, and suppresses cGAS-STING–AIM2-mediated neuroinflammation, suggesting its therapeutic potential for HIV- and opioid-associated CNS dysfunction (Fig. 8H).

## Discussion

HAND continues to affect a significant proportion of PLWH, even with effective cART, underscoring that viral suppression alone does not prevent neurocognitive decline [53, 54]. Increasing evidence implicates persistent low-level viral protein expression, including HIV-1 Tat, and comorbid factors like opioid abuse as major contributors to chronic neuroinflammation and neuronal injury [1, 3–5]. HIV-Tat protein disrupts neuronal and glial function through oxidative stress, mitochondrial damage, and epigenetic dysregulation, while opioids intensify these effects by promoting microglial activation, impairing neuroprotective signaling, and altering chromatin structure [40, 50, 55, 56].

Beyond transcriptional dysregulation, accumulating evidence indicates that genomic instability and impaired DDR mechanisms play central roles in HAND pathology [26]. Persistent DNA DSBs, impaired repair machinery, and activation of DDR kinases such as ATM initiate proinflammatory cascades, including NF-κB-mediated cytokine expression and cGAS–STING-dependent interferon signaling, leading to chronic neuroinflammation [57–61]. Our study integrates data from human microglial models, iTat transgenic mice, and postmortem HIV-positive brain tissue to delineate how HIV-Tat protein and opioids converge to impair DNA repair, chromatin organization, and neuroimmune balance. In vitro, acute HIV-Tat exposure promptly activated canonical DDR signaling, as evidenced by increased γH2AX, phosphorylated ATM (Ser1981), and NBS1 and 53BP1 expression. Chronic Tat protein exposure amplified DDR activation, and morphine co-treatment further enhanced these responses, suggesting that opioids potentiate viral protein-induced genotoxic stress [18, 62]. The engagement of error-prone non-homologous end-joining (NHEJ) repair proteins, such as 53BP1 and NBS1, implies preferential activation of imprecise repair processes under sustained stress, potentially contributing to mutation accumulation [63]. Upregulation of KAT5 (TIP60) in the morphine and co-treated groups indicates an adaptive chromatin remodeling response to persistent genotoxicity, consistent with Tip60’s role in coupling chromatin stability with DDR [64, 65].

Consistent with these findings, chronic exposure to Tat protein and morphine altered key components of the NER pathway in microglia. ERCC2 and ERCC3 were significantly downregulated, whereas ERCC6 expression was increased, suggesting activation of compensatory transcription-coupled repair mechanisms. Notably, acute exposure had no effects on NER proteins, underscoring the temporal dynamics of DNA repair dysfunction in microglial cells. These results align with prior studies that have linked ERCC deficiencies to elevated oxidative stress, microglial activation, and a senescence-like phenotype [28, 66], supporting the recruitment of chromatin remodelers in response to sustained DNA damage. Therefore, chronic exposure models were prioritized for subsequent analyses.

In addition, BRG1 induction during chronic Tat and morphine exposure indicates recruitment of the SWI/SNF chromatin-remodeling complex to damaged loci, establishing a mechanistic link between DNA repair deficits and epigenetic reorganization. BRG1/ARID1A, a critical SWI/SNF subunit, was similarly upregulated, consistent with compensatory chromatin restructuring to facilitate repair and transcriptional adaptation. Together, these findings suggest that HIV-Tat and morphine disrupt LOCK chromatin integrity, shifting the landscape from a repressive toward a more transcriptionally permissive state, thereby exacerbating genomic instability and inflammatory signaling. These observations are consistent with our previous work, showing that HIV gp120 and Bal virus activate the SWI/SNF and ARID1A pathways, further reinforcing the connection between viral signaling and chromatin destabilization [5, 67, 68].

Chromatin organization is a key determinant of genome stability, and disruption of LOCKs has been linked to widespread transcriptional and DNA repair defects. Co-exposure to Tat protein and morphine reduced repressive histone marks (H3K9me3, H3K27me3) while increasing activating marks (H3K9ac, H3K27ac), indicating a shift toward chromatin relaxation and transcriptional permissiveness. This remodeling likely amplifies DDR impairment [69] by promoting aberrant expression of inflammatory genes while restricting access of repair factors to damaged regions [70]. H2A.X knockdown experiments further confirmed that ATM is central to coordinating DDR-driven chromatin responses and neuroimmune signaling. Specifically, H2A.X silencing in microglia reduced γH2AX accumulation, ATM and NBS1 phosphorylation, restored ERCC2 expression, and suppressed AIM2 inflammasome activation, demonstrating its dual role as a DNA damage sensor and transcriptional regulator [71, 72]. These findings suggest that selective ATM modulation may interrupt the cycle of DNA damage, chromatin relaxation, and neuroinflammatory amplification in HAND, supporting previous observations that HIV proteins and opioids dysregulate inflammasome pathways [40].

In parallel, microglia exposed to HIV-Tat and morphine release immune mediators that reduce neuronal viability, indicating that activated microglia worsen HIV-Tat-induced neurodegeneration [7, 73]. Combined exposure enhances inflammatory signaling and disrupts neuronal DDRs, including altered chromatin marks and reduced ATM and AIM2 expression. Notably, H2A.X silencing in neurons co-cultured with microglia subjected to H2A.X knockdown and exposed to Tat and morphine attenuated ATM and AIM2 signaling and mitigated neuronal toxicity, highlighting the critical role of microglia-neuron crosstalk in shaping neuronal vulnerability.

Extending these findings in vivo, iTat transgenic mice subjected to a 4-day escalating morphine regimen exhibited robust DDR activation, evidenced by increased γH2AX, total and phosphorylated ATM, and NBS1 levels in cortical tissue. Morphine co-administration further enhanced these markers, mirroring the synergistic effects observed in vitro. Dysregulation of chromatin remodelers, including ARID1A and BRG1, indicated compromised SWI/SNF complex function and impaired nucleosome repositioning. Histone modification analyses confirmed heterochromatin erosion, with decreased H3K9me3 and increased H3K9ac, consistent with a persistent open-chromatin state that predisposes to transcriptional instability. Evaluation of NER genes revealed selective activation of ERCC1 and ERCC8 but downregulation of ERCC2, ERCC3, and ERCC6, suggesting incomplete or inefficient repair under Tat + morphine exposure. These results align with studies showing that chronic genotoxic stress drives heterochromatin remodeling through altered H3K9me3 dynamics [74] and highlight multiple strategies human pathogens use to manipulate DDR pathways. Collectively, our findings indicate that targeted modulation of DDR components may represent a promising therapeutic strategy for mitigating HIV-associated neuroinflammation.

Furthermore, activation of KAT5/Tip60 alongside the cGAS–STING–AIM2 inflammasome axis in iTat mouse brains highlights a coordinated mechanism linking genotoxic stress to innate immune activation. This response was most pronounced under combined Tat and morphine exposure, indicating that opioids amplify Tat-induced DNA damage signaling [18, 61]. The resulting accumulation of cytosolic DNA acts as a danger signal that engages STING, triggering a classical antiviral program. STING activation subsequently drives NF-κB-dependent proinflammatory signaling, leading to the production of cytokines that are individually and synergistically neurotoxic. Consistent with this, we show that these inflammatory mediators are sufficient to reduce neuronal viability in vivo.

Although prior studies have linked the DDR to inflammatory pathways, such as the cGAS–STING axis [75], this relationship has been less explored in the context of inflammasome biology. Our findings reveal that NLRP3 directly augments DDR signaling, independent of its canonical inflammasome function. Specifically, NLRP3 enhances ATM autophosphorylation and promotes phosphorylation of key DDR effectors, including H2AX, CHK2, and p53, the latter of which contributes to apoptosis [76].

Therapeutically, DMF, a clinically approved antioxidant and neuroprotective agent, attenuated Tat + morphine-induced genotoxicity in vivo. DMF administration reduced ATM phosphorylation, normalized NBS1 and ERCC1 expression, and restored H3K9me3-mediated repression, suggesting partial repair of LOCK chromatin architecture. In parallel, DMF significantly suppressed activation of the cGAS–STING–AIM2 inflammasome axis and reduced downstream production of proinflammatory cytokines, highlighting its capacity to mitigate Tat- and morphine-induced neuroinflammation. These findings are consistent with prior reports demonstrating that DMF limits oxidative DNA damage, microglial activation, and neuronal degeneration in models of multiple sclerosis and viral neuroinflammation [77–80]. Importantly, our results align with growing evidence that DMF may protect against both systemic and CNS complications of HIV infection [81]. Notably, a recent study by Kolson and colleagues repurposed DMF for use in SIV-infected rhesus macaques, where it reduced brain oxidative stress, findings the authors interpreted as potentially inducing neuroprotective responses in persons living with HIV [49, 82].

Moreover, our behavioral analyses demonstrated that DOX-induced mice expressing the Tat protein exhibited significant impairments in NOR. These findings align with our previous studies using iTat mice [47, 52, 83] and support earlier reports showing that exogenous administration of Tat protein directly into the brain disrupts spatial learning and memory, including performance in the eight-arm radial maze [84] and increased latency to locate the hidden platform in the Morris water maze [85]. Such deficits in learning and memory have been attributed to Tat-induced hippocampal synaptic dysfunction [10] and dysregulation of striatal dopamine transporters and catecholamine levels [83]. Extending these observations, we confirm here that the Tat protein alone is sufficient to impair NOR, as demonstrated in Tat-null mice. Notably, repeated morphine exposure over 4 days produced comparable deficits in both iTat and Tat-null mice, consistent with prior evidence that opioid exposure negatively affects learning and memory in NOR tasks^86^, as well as decision-making and spatial learning paradigms [87].

Importantly, and consistent with our mechanistic findings, prophylactic daily administration of DMF ameliorated Tat-induced deficits in NOR. Although these initial behavioral results would benefit from further dose–response and temporal characterization, they are broadly aligned with increasing evidence that DMF mitigates memory impairments across multiple models of neurodegeneration, including rodents exposed to streptozotocin [88], isoflurane-induced neurotoxicity, and several transgenic models of Alzheimer’s disease [89, 90]. Taken together, these results indicate that DMF has therapeutic potential to mitigate HIV-associated neurocognitive impairment, though further studies are required to fully validate its efficacy and extend these behavioral findings.

Validation studies using postmortem HIV-positive frontal cortical tissue confirmed increased γH2AX accumulation and reduced ERCC2 and ERCC3 expression, mirroring the signatures identified in both our in vitro and in vivo models. These findings underscore the translational relevance of our observations and illustrate how viral protein expression and opioid exposure converge to impair genome maintenance and promote neuroinflammation in HAND pathology. Collectively, the data define a mechanistic framework in which HIV-Tat and opioids synergistically activate the KAT5–ATM–cGAS–STING signaling axis, driving persistent DNA damage, chromatin remodeling deficits, and microglial-associated inflammation. In vitro, H2A.X emerges as a central integrator of DDR and innate immune responses, while in vivo co-exposure to Tat and morphine recapitulates the full cascade of genotoxic stress, epigenetic dysregulation, and inflammasome activation. Importantly, DMF demonstrates significant therapeutic potential by restoring genomic stability, reinforcing heterochromatin structure, and suppressing inflammatory signaling.

The convergence of DNA damage, epigenetic disruption, and neuroimmune activation highlights DDR–chromatin crosstalk as a pivotal driver of HAND. Future studies should focus on cell-type-specific DDR dynamics within microglia vs astrocytes and evaluate the long-term efficacy of DMF, particularly its effects on neuronal connectivity, synaptic resilience, and cognition. Multi-omics integration will be essential for further delineating how redox imbalance, chromatin remodeling, and DDR signaling intersect to govern neurodegenerative trajectories in HAND. Therapeutically, strategies aimed at restoring ARID1A-dependent chromatin accessibility and attenuating ATM–cGAS–STING-mediated inflammation may provide broad neuroprotection in PLWH with opioid comorbidities.

In conclusion, our findings demonstrate that HIV-Tat and morphine, both independently and synergistically, disrupt DNA repair and chromatin integrity via the KAT5–ATM–cGAS–STING pathway, leading to persistent genotoxic stress, heterochromatin erosion, and heightened inflammatory activation. Importantly, H2A.X silencing and DMF treatment partially restored DNA repair capacity, reinforced chromatin structure, and attenuated inflammasome signaling, highlighting their potential as therapeutic interventions. These results provide a mechanistic framework for targeting DDR–chromatin crosstalk to mitigate HIV–opioid–associated neuroinflammation and cognitive decline in HAND.

### Institutional review board statement

Human brain tissue specimens from HIV-positive individuals and uninfected controls, including frontal and temporal cortex samples, were obtained from the NNTC and the NNAB, David Geffen School of Medicine at the University of California, Los Angeles (UCLA), USA. All human tissue collections were performed under protocols approved by the respective Institutional Review Boards (IRBs), and the repositories provided de-identified specimens for research in accordance with applicable ethical guidelines and regulations.

### Informed consent statement

Not applicable. The study used de-identified human brain tissue obtained from established biorepositories (NNTC and NNAB). Informed consent was obtained by the tissue repositories at the time of specimen collection in accordance with their Institutional Review Board-approved protocols.

## Supplementary information

CDD-Karthick et al_Rev_Supplementary Table 1_Final_6th July_2026.docx

CDD-Karthick et al_Rev_Supplementary File_uncropped blot images_Final 6th July_2026.pdf

## Data Availability

The datasets generated and/or analyzed during the current study are available from the corresponding author upon reasonable request.
