# Supplementary material for: Epigenetic LOCK chromatin links H3K9 modifications to ATM–γH2AX signaling in HIV-Tat and opioid neuroinflammation
Source: Cell Death Dis. 2026 Aug 5;17(1):932. doi: 10.1038/s41419-026-09146-6 (PMC13728189; doi:10.1038/s41419-026-09146-6)
Supplement: Supplementary file 1 [file 41419_2026_9146_MOESM2_ESM.pdf]

Epigenetic rewiring of LOCK chromatin Links H3K9 modifications to ATM-γH2AX signaling in HIV-Tat and opioid neuroinflammation: Insights from HIV-positive brain

Karthick Chennakesavan[1], Malar Samikkannu[1], Haylee R. Hammond[2], Shainnel O. Eans[2], Marc J. Kaufman[3], Jay P. McLaughlin[2], and Thangavel Samikkannu[1*]

[1]Department of Pharmaceutical Sciences, Irma Lerma Rangel College of Pharmacy, Texas A&M, University, College Station, Texas- 77843, United States of America.

[2]Department of Pharmacodynamics, College of Pharmacy, University of Florida, Gainesville, Florida-32610, United States of America.

[3]McLean Imaging Center, McLean Hospital/Harvard Medical School, Belmont, Massachusetts-02478, United States of America.

*Corresponding Author:

Thangavel Samikkannu, Ph.D.
Associate Professor
Department of Pharmaceutical Sciences
Irma Lerma Rangel College of Pharmacy
Texas A&M University
College Station
Texas-77845, USA.
Tel: 979-436-9916
Fax: 979-436-0087
Email: thangavel@tamu.edu

Supplementary Figures

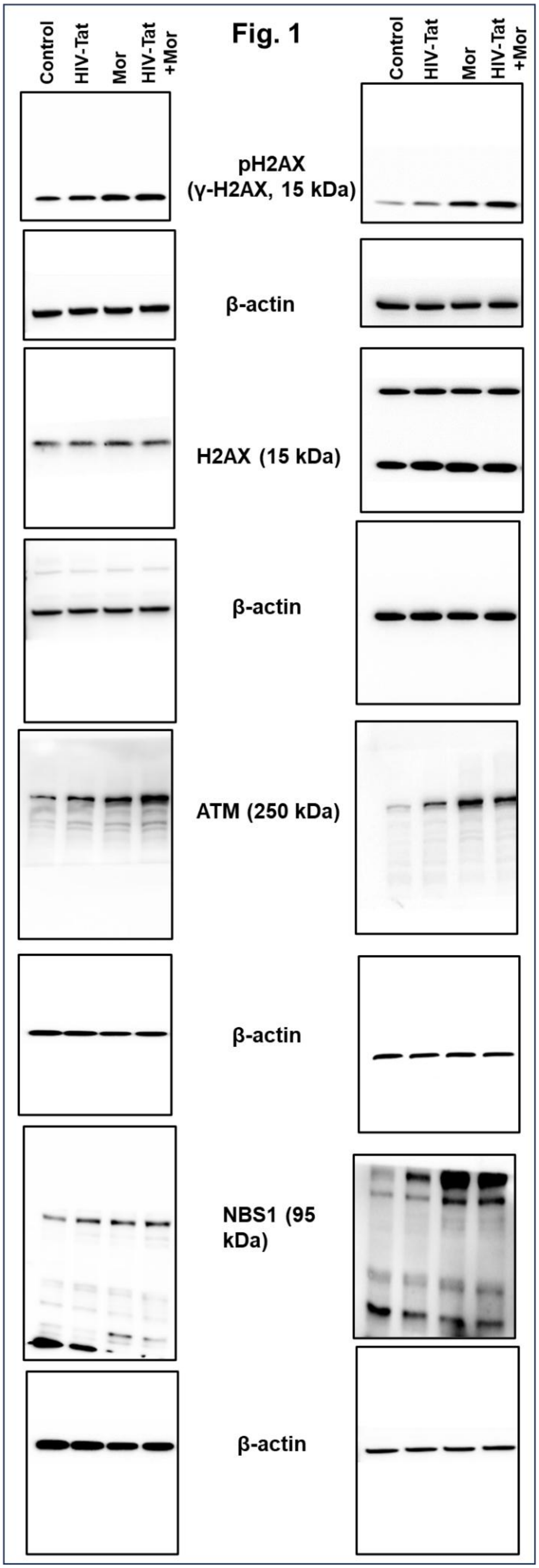

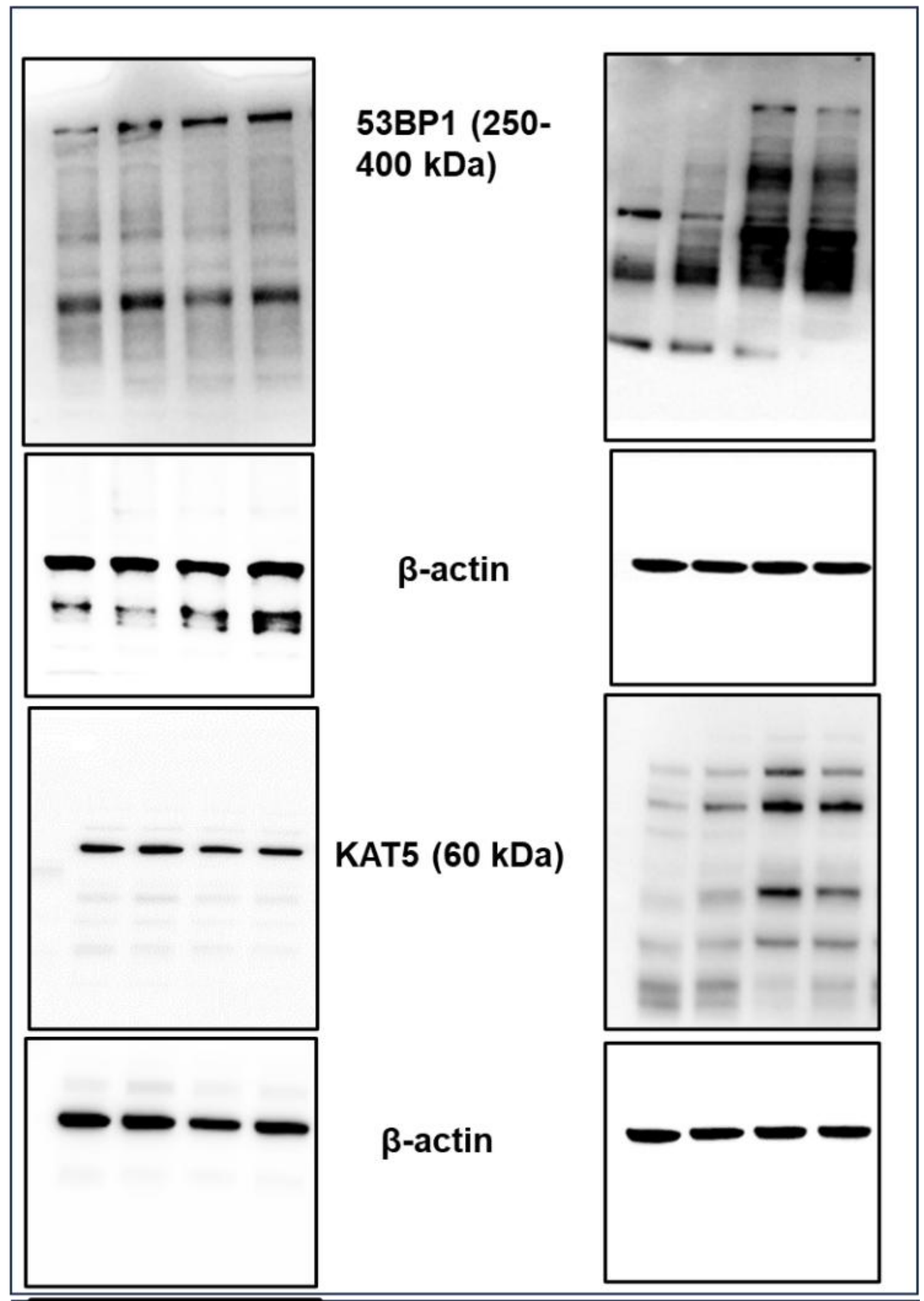

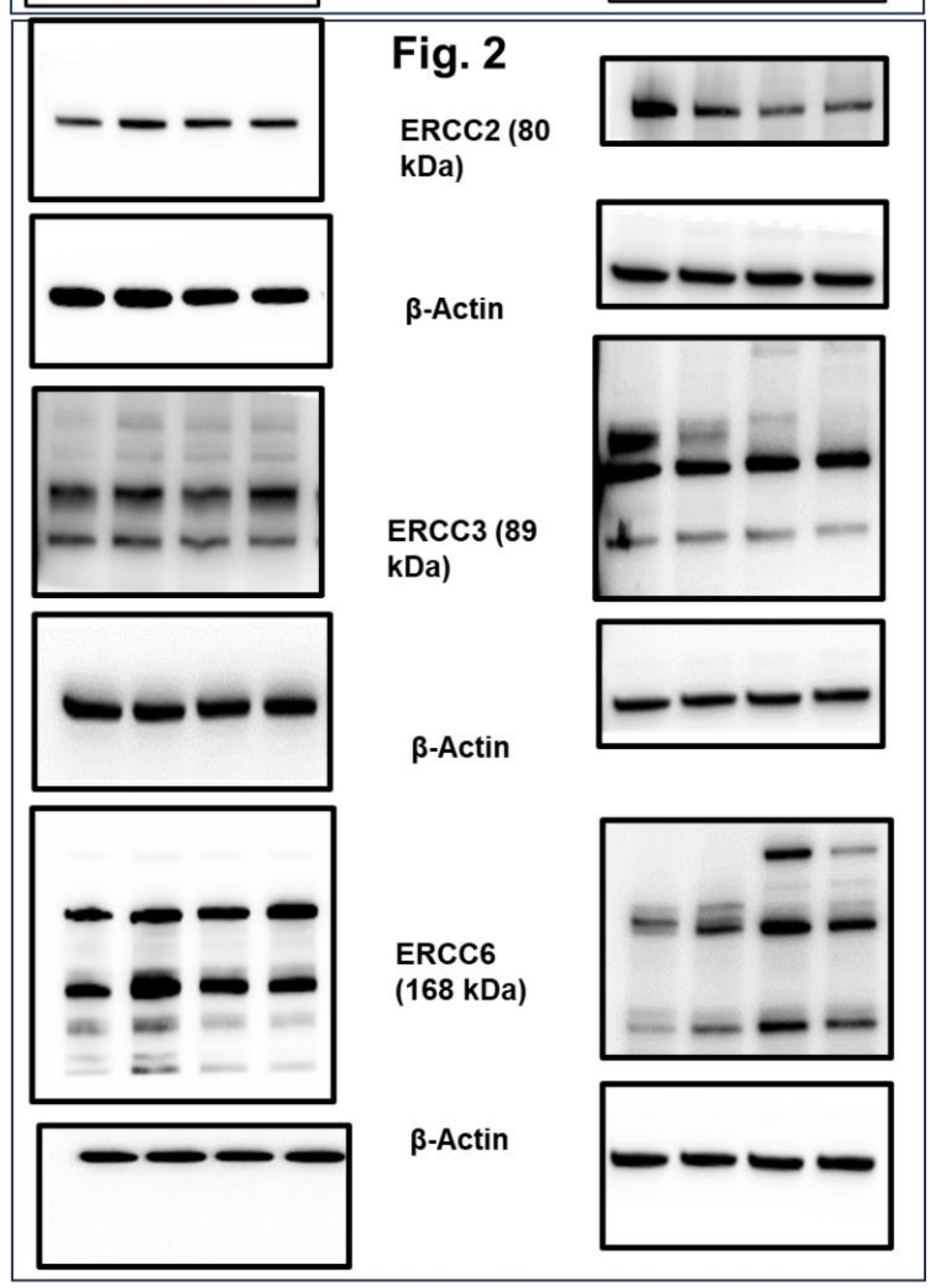

Fig. 2

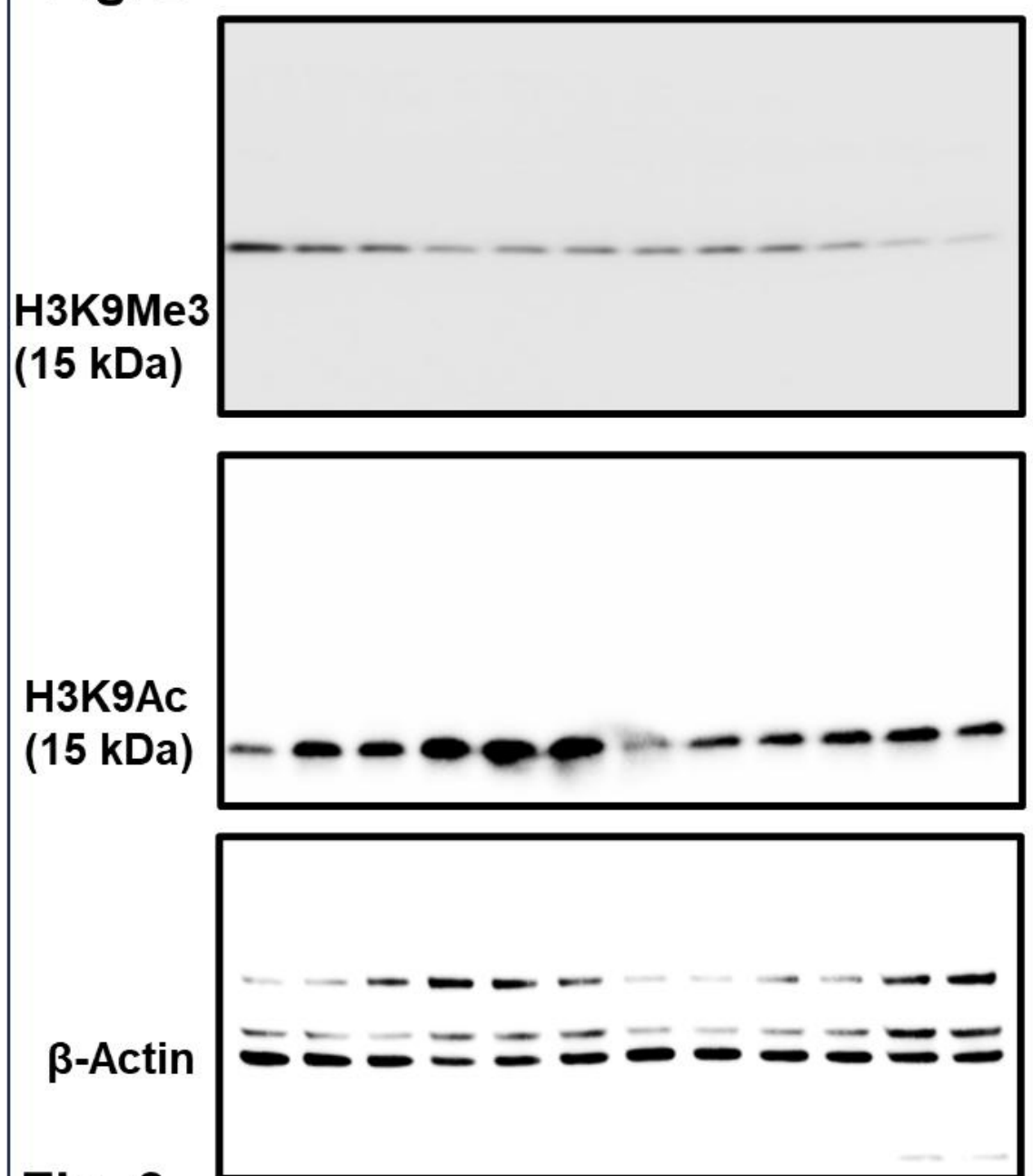
H3K9Me3 (15 kDa)
H3K9Ac (15 kDa)
β-Actin

Fig. 3

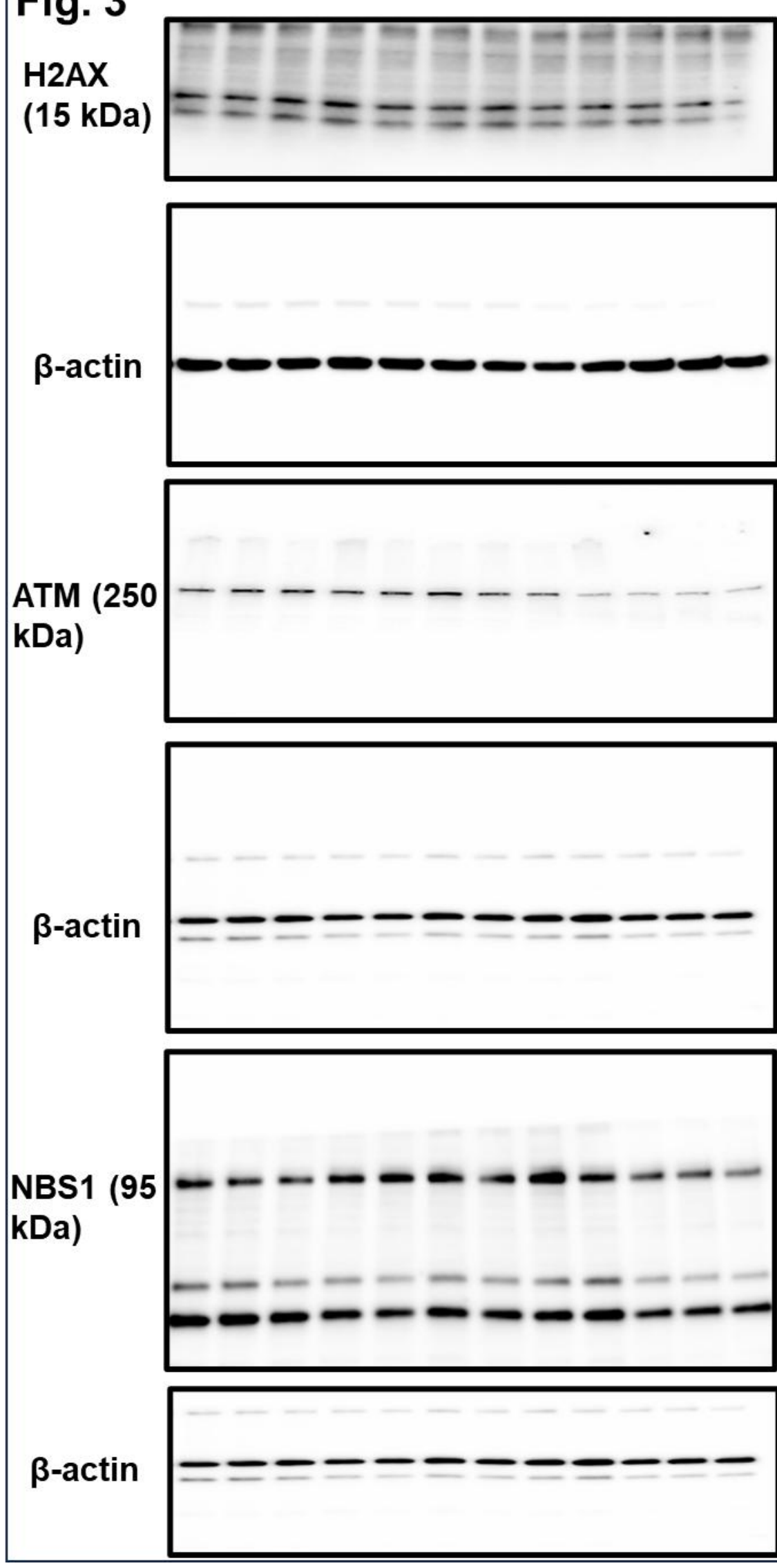
H2AX (15 kDa)
β-actin
ATM (250 kDa)
β-actin
NBS1 (95 kDa)
β-actin

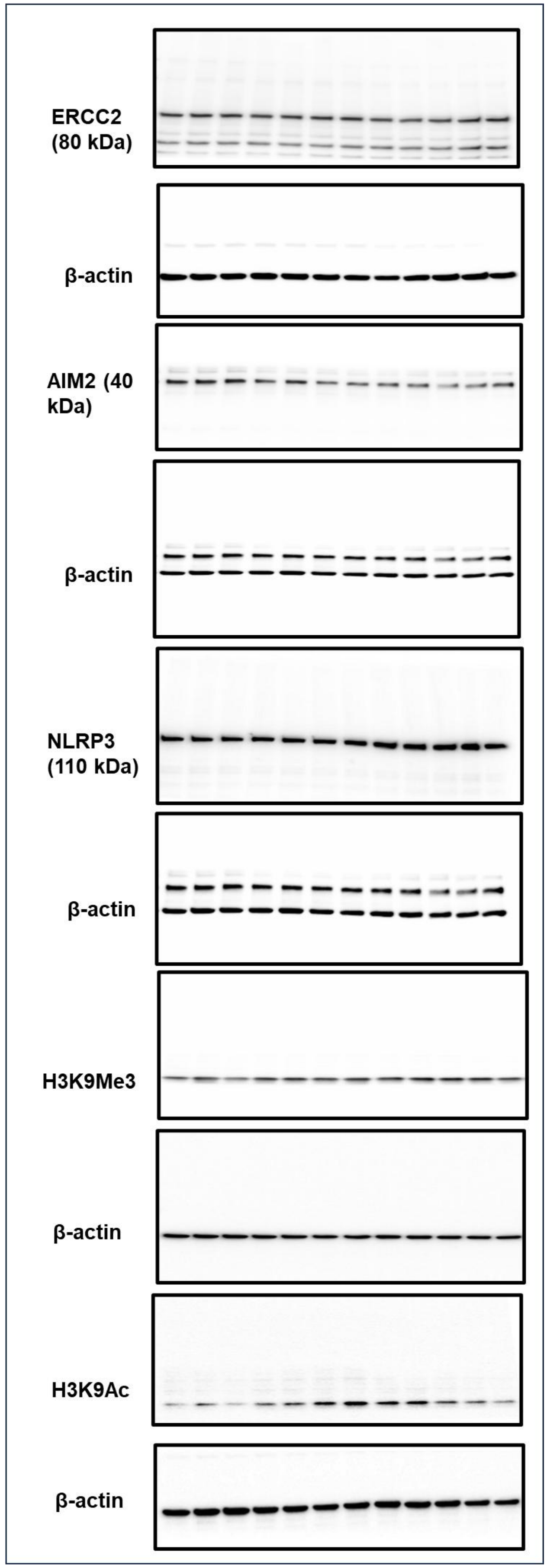
ERCC2 (80 kDa)
β-actin
AIM2 (40 kDa)
β-actin
NLRP3 (110 kDa)
β-actin
H3K9Me3
β-actin
H3K9Ac
β-actin

Fig. 4

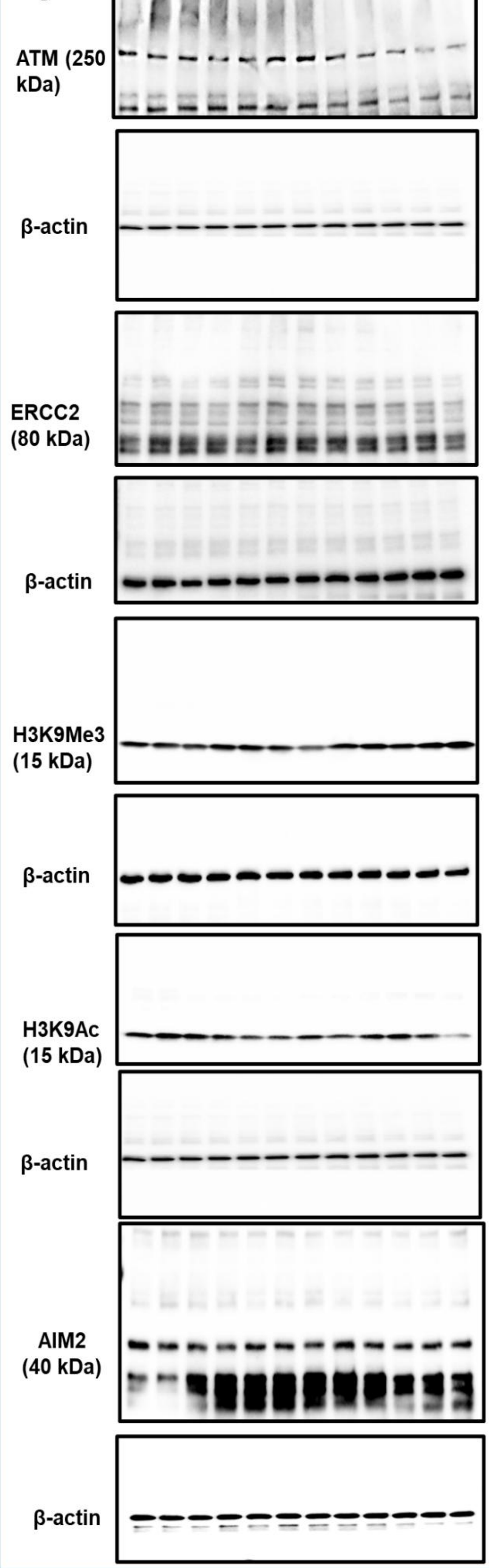
ATM (250 kDa)
β-actin
ERCC2 (80 kDa)
β-actin
H3K9Me3 (15 kDa)
β-actin
H3K9Ac (15 kDa)
β-actin
AIM2 (40 kDa)
β-actin

Fig. 5

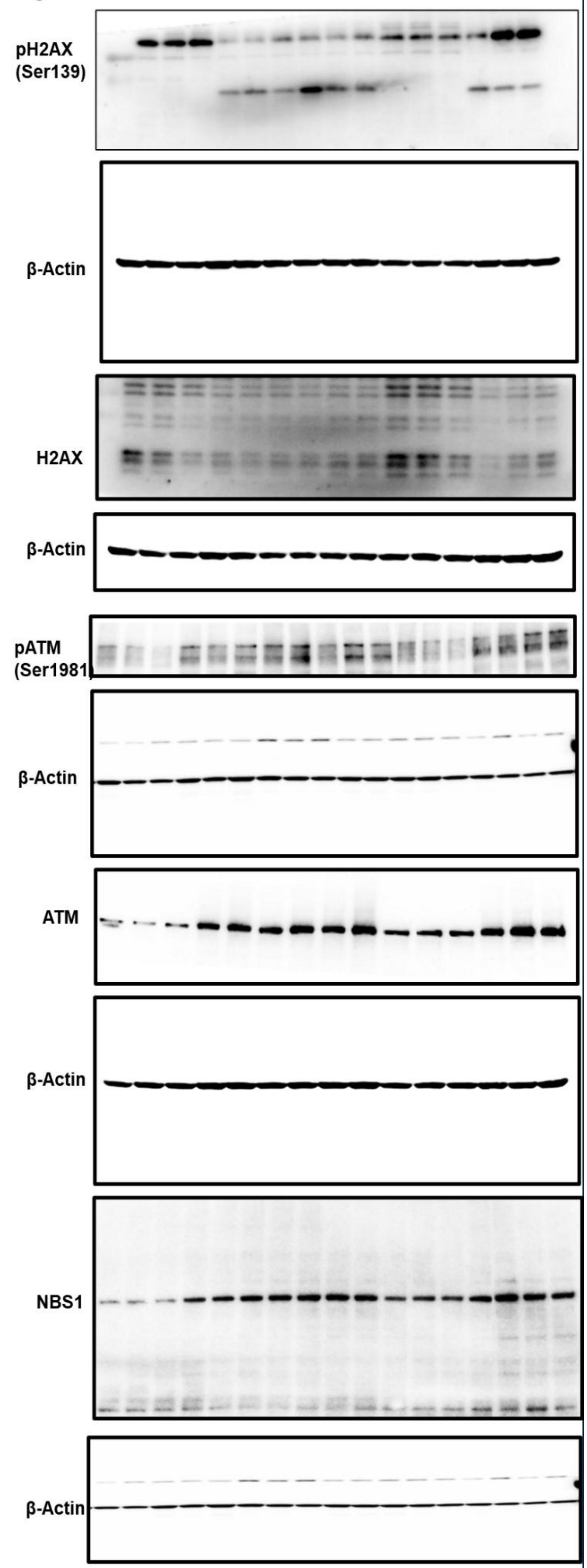
pH2AX (Ser139)
β-Actin
H2AX
β-Actin
pATM (Ser1981)
β-Actin
ATM
β-Actin
NBS1
β-Actin

Fig. 5

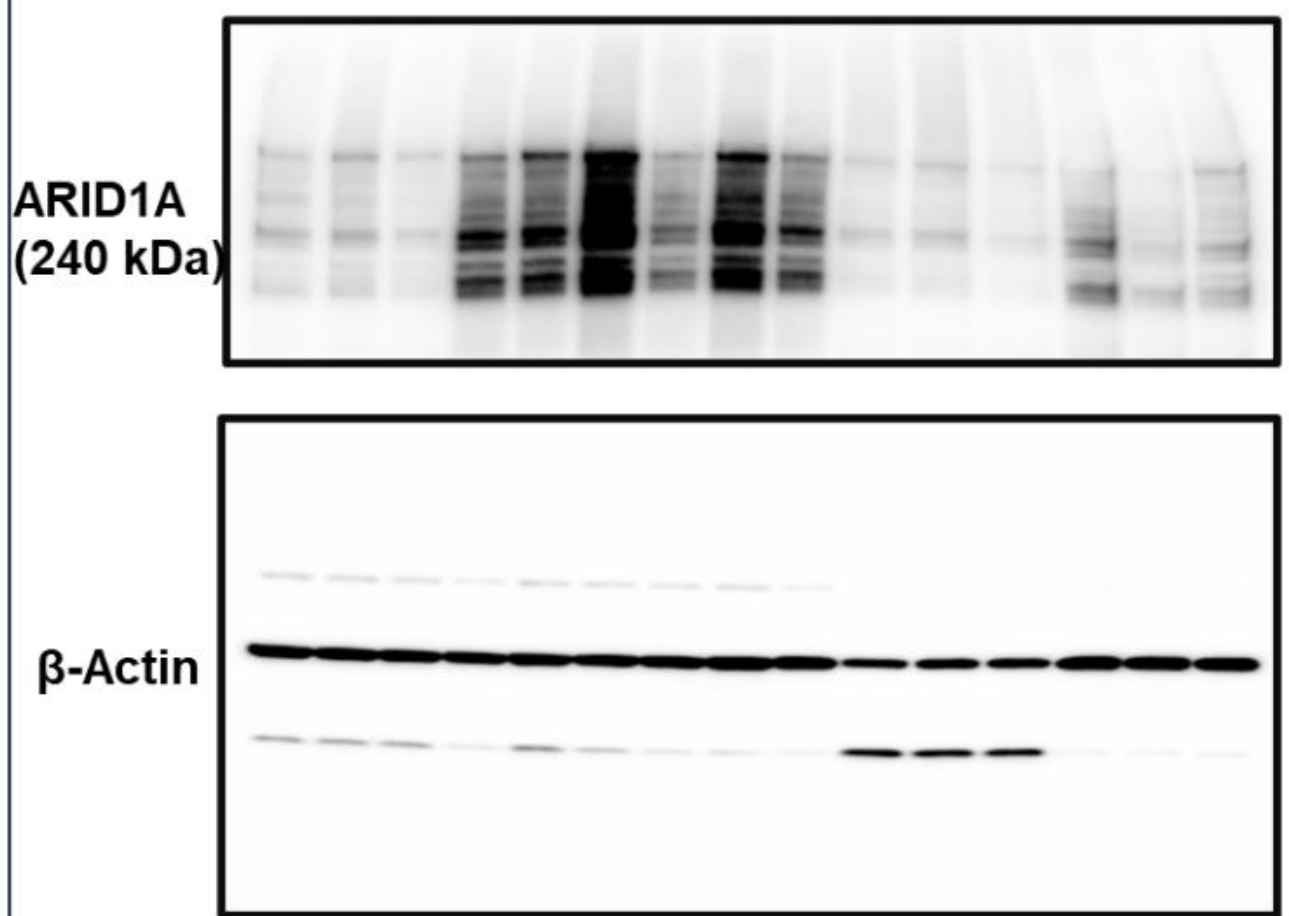

Fig. 6

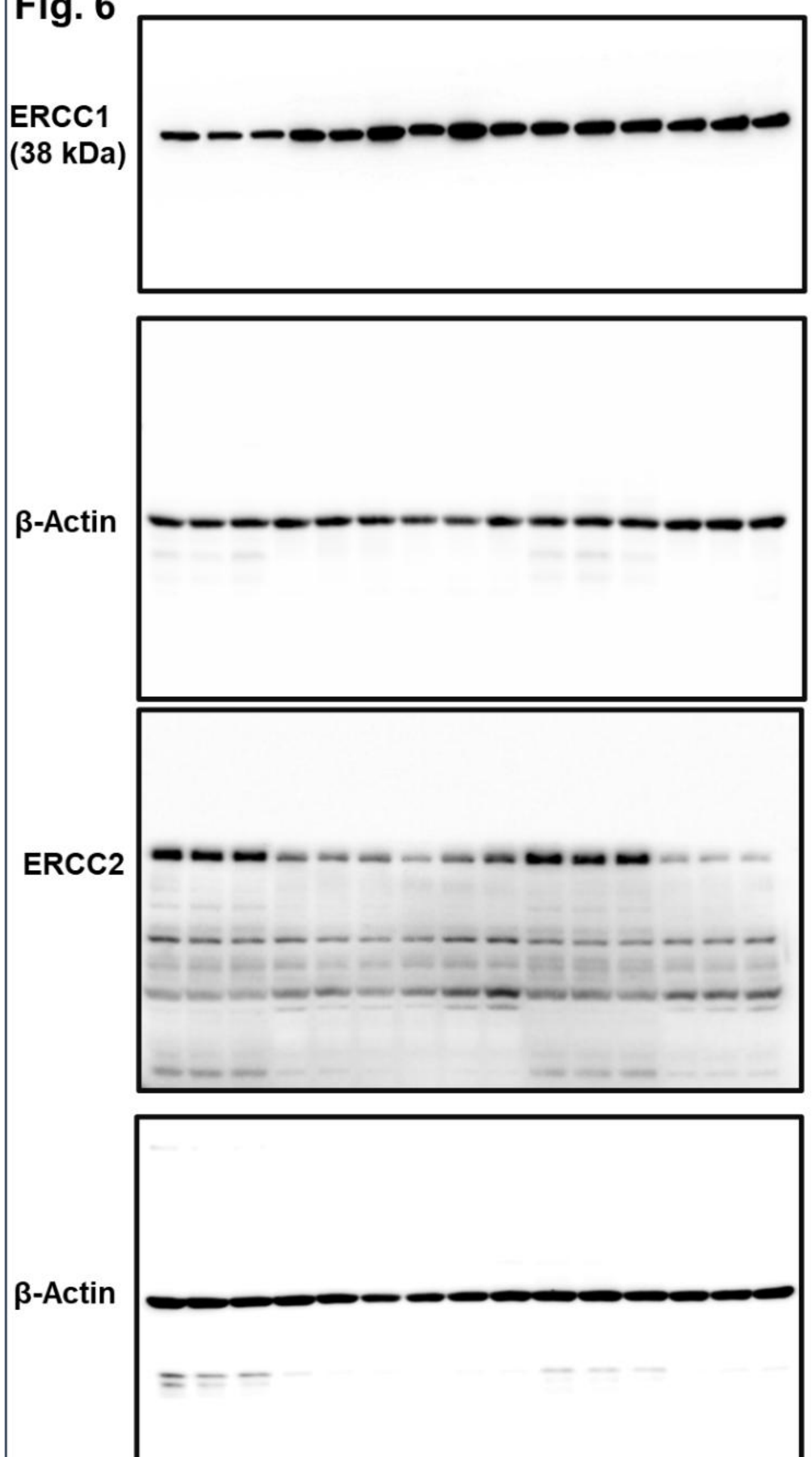

Fig. 5

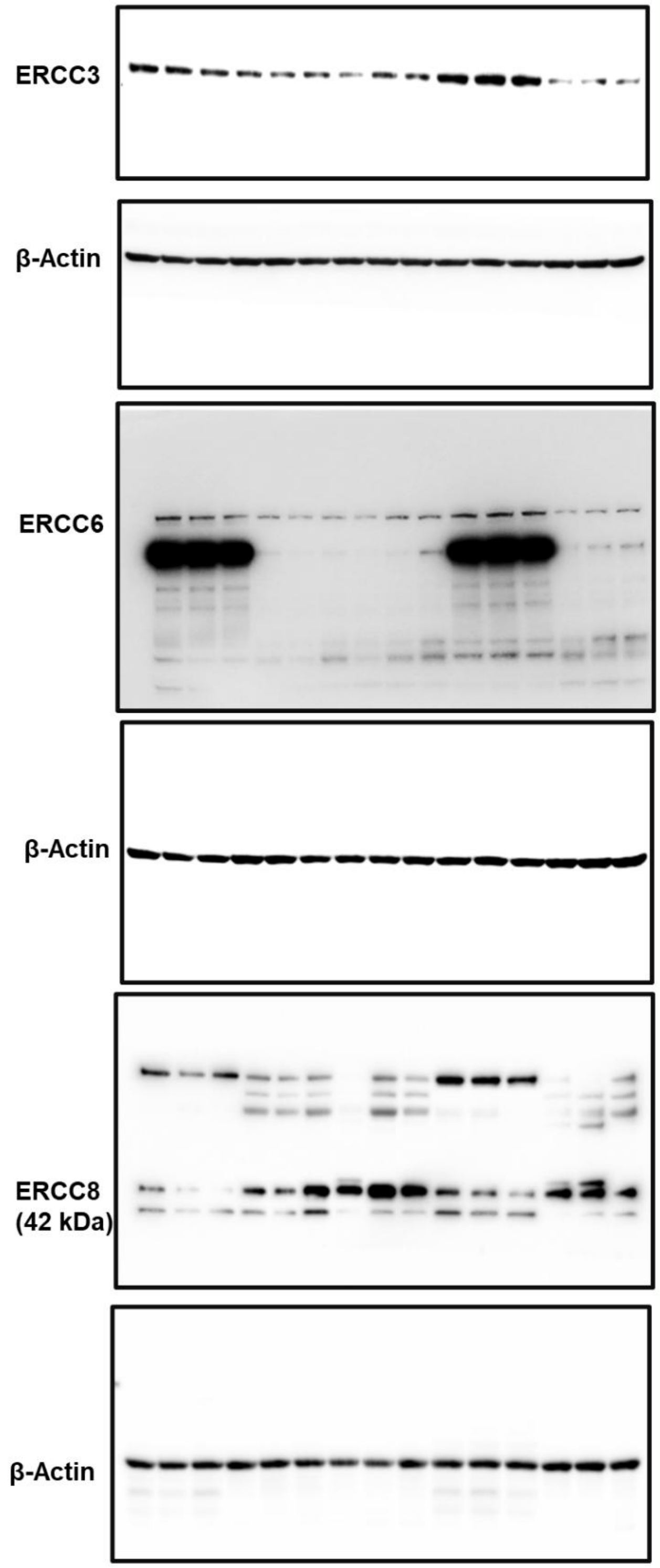

Fig. 5

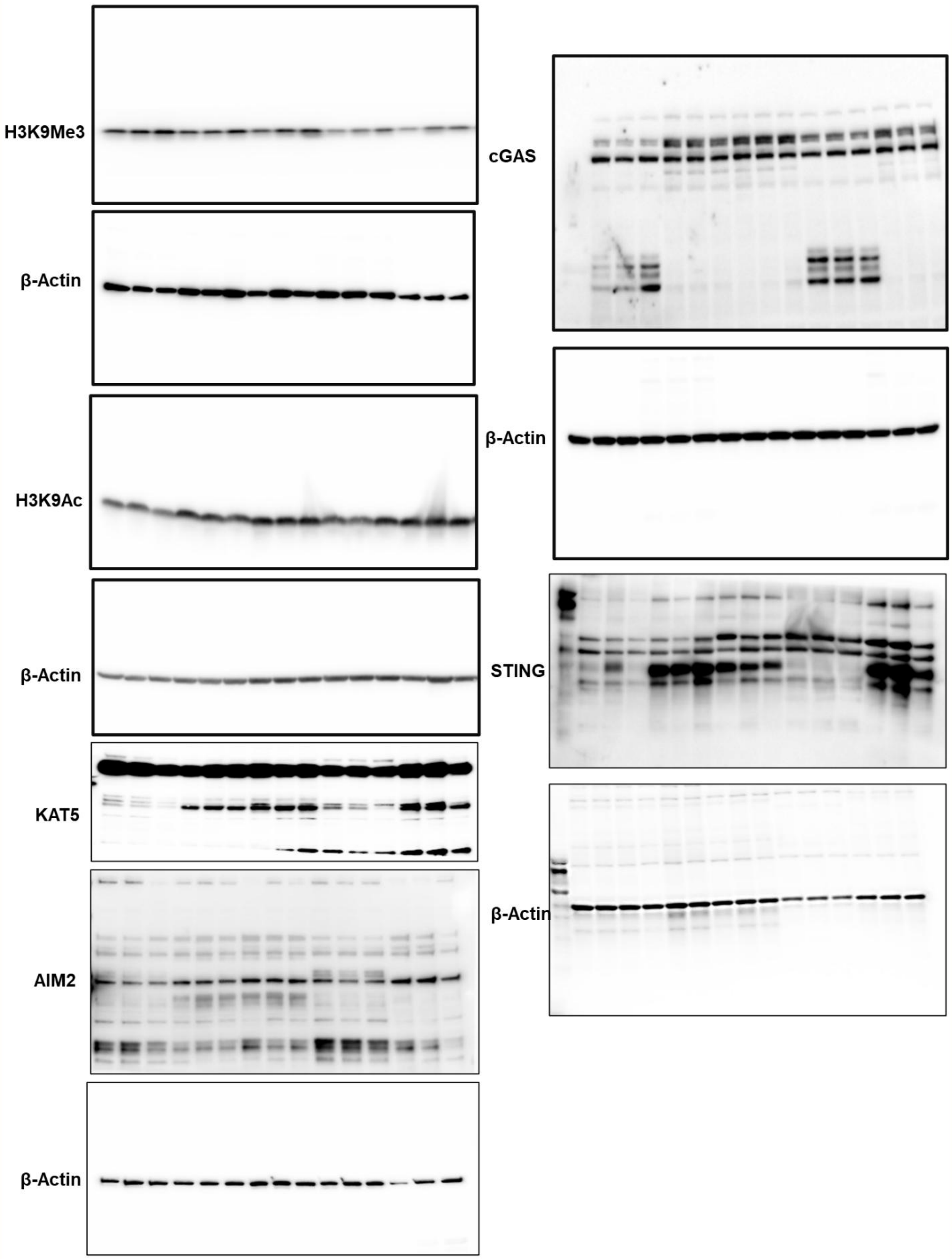
H3K9Me3
β-Actin
H3K9Ac
β-Actin
KAT5
AIM2
β-Actin
cGAS
β-Actin
STING
β-Actin

Fig. 6

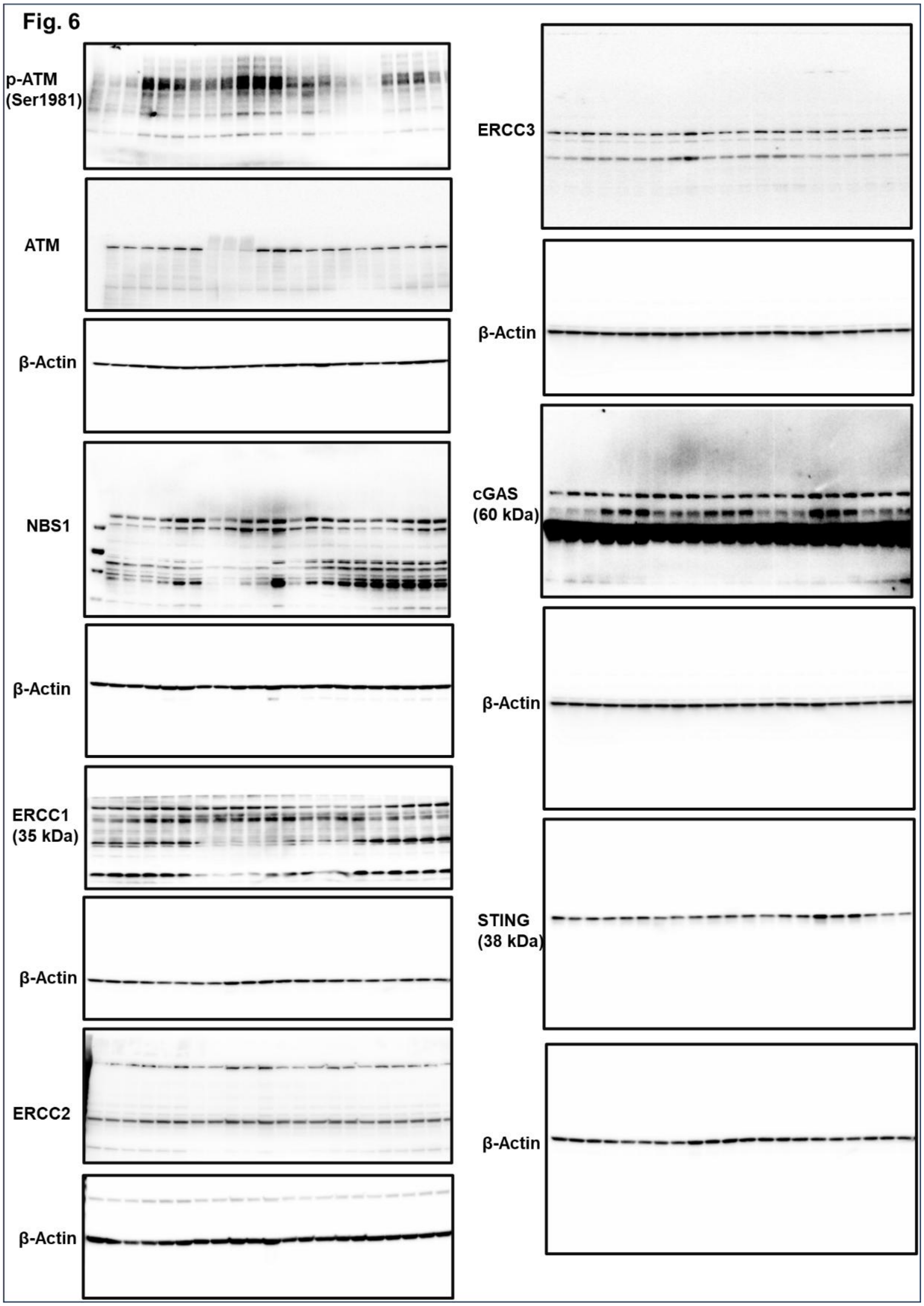

p-ATM (Ser1981)
ATM
β-Actin
NBS1
β-Actin
ERCC1 (35 kDa)
β-Actin
ERCC2
β-Actin
ERCC3
β-Actin
cGAS (60 kDa)
β-Actin
STING (38 kDa)
β-Actin

Fig. 6

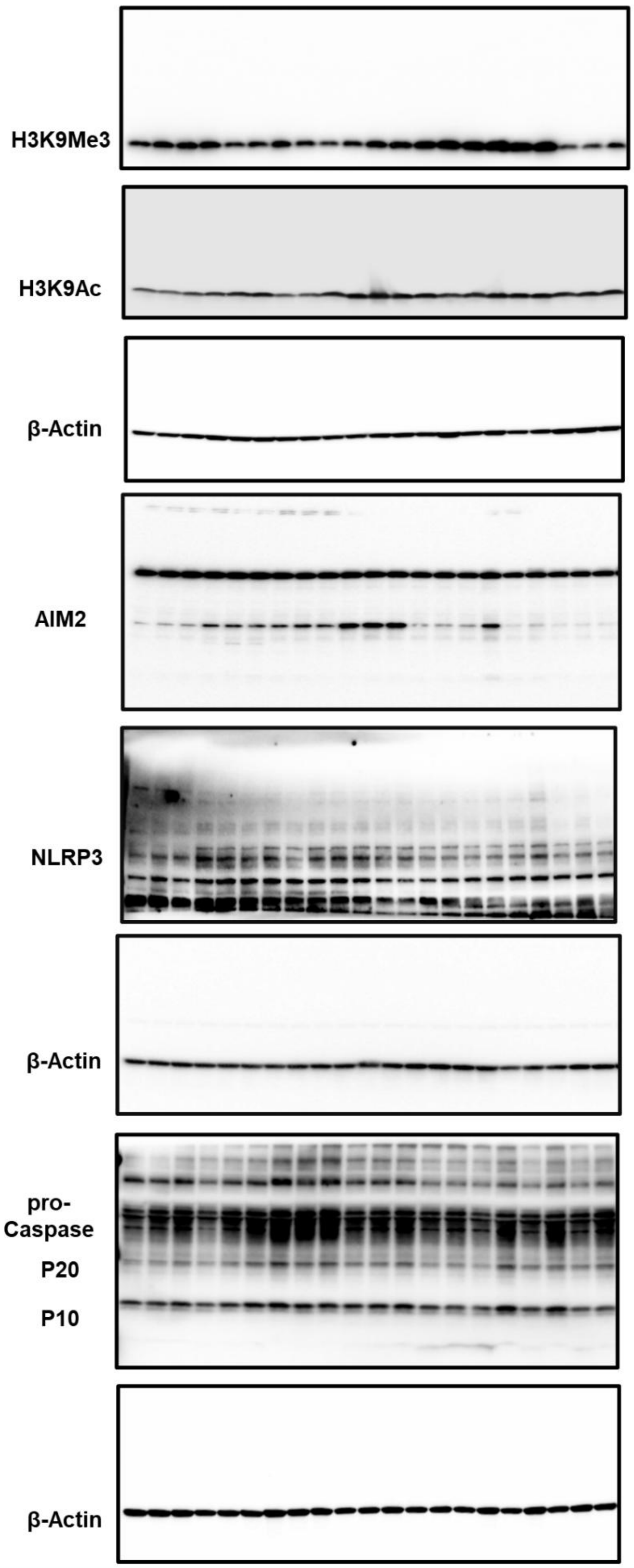
H3K9Me3
H3K9Ac
β-Actin
AIM2
NLRP3
β-Actin
pro-
Caspase
P20
P10
β-Actin

Fig. 8

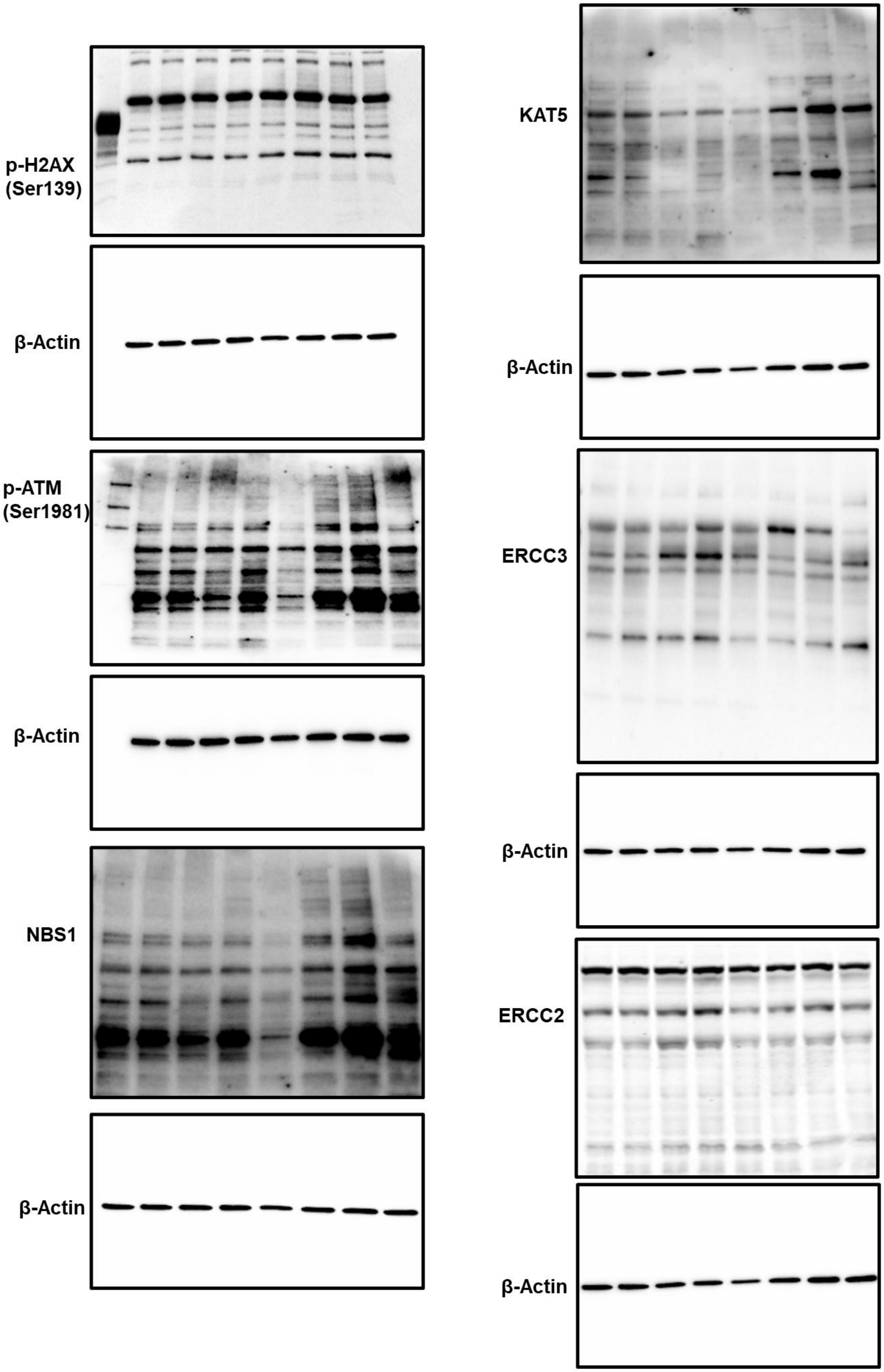

p-H2AX
(Ser139)
β-Actin
p-ATM
(Ser1981)
β-Actin
NBS1
β-Actin
KAT5
β-Actin
ERCC3
β-Actin
ERCC2
β-Actin
