# Supplementary material for: Epigenetic LOCK chromatin links H3K9 modifications to ATM–γH2AX signaling in HIV-Tat and opioid neuroinflammation
Source: Cell Death Dis. 2026 Aug 5;17(1):932. doi: 10.1038/s41419-026-09146-6 (PMC13728189; doi:10.1038/s41419-026-09146-6)
Supplement: Supplementary file 2 [file 41419_2026_9146_MOESM1_ESM.docx]

**Epigenetic rewiring of LOCK chromatin Links H3K9 modifications to ATM-γH2AX signaling in HIV-Tat and opioid neuroinflammation: Insights from HIV-positive brain**

**Karthick Chennakesavan^1^, Malar Samikkannu^1^, Haylee R. Hammond^2^, Shainnel O. Eans^2^, Marc J. Kaufman^3^, Jay P. McLaughlin^2^, and Thangavel Samikkannu^1*^**

*^1^Department of Pharmaceutical Sciences, Irma Lerma Rangel College of Pharmacy, Texas A&M, University, College Station, Texas- 77843, United States of America.*

*^2^Department of Pharmacodynamics, College of Pharmacy, University of Florida, Gainesville, Florida-32610, United States of America.*

*^3^McLean Imaging Center, McLean Hospital/Harvard Medical School, Belmont,* [*Massachusetts*](https://www.google.com/search?q=Belmont%2C+Massachusetts&sca_esv=7f3205b42edbd4d3&hl=en&sxsrf=AE3TifPfo_-lnuIeoV70y2awpCNwJf4Z5Q%3A1765071714226&source=hp&ei=Yts0afaMC4-nmtkPtJD1mQg&iflsig=AOw8s4IAAAAAaTTpcuIg5dsaDYGk-rsCtmgnwCDBB81G&ved=2ahUKEwjghbPGrKqRAxW1kmoFHWh-J7QQgK4QegQIARAE&oq=MA+02478&gs_lp=Egdnd3Mtd2l6IghNQSAwMjQ3ODIFECEYoAEyBRAhGKABMgUQIRigATIFECEYoAFIgQxQAFgAcAB4AJABAJgBa6ABa6oBAzAuMbgBDMgBAPgBAvgBAZgCAaACcZgDAOIDBRIBMSBAkgcDMC4xoAeSArIHAzAuMbgHccIHAzAuMcgHAoAIAA&sclient=gws-wiz&mstk=AUtExfBh1MtwEz2um6PQmJ6Nbmi5mSQy3WoBQxfUp2ZCXSnVh86icu1ScGGA8BAkS-HerY0Y-NbY5sISbqwKYYua8TVZyVrpUmlGmVL4r3mogVXck1YWhRylCCscc1RTmhYb9JNVWNrclGb1QeWRvpTiNqhDTnVkimQf-4N0hooQqmy32PQ&csui=3)*- 02478, United States of America.*

*Corresponding Author:

Thangavel Samikkannu, Ph.D.

Associate Professor

Department of Pharmaceutical Sciences

Irma Lerma Rangel College of Pharmacy

Texas A&M University

College Station

Texas-77845, USA.

Tel: 979-436-9916

Fax: 979-436-0087

Email: thangavel@tamu.edu

**Supplementary Table 1**: List of Antibodies used for Western blot analysis and Immunohistochemistry

| **Antibody** | **Company** | **Cat #** |
| --- | --- | --- |
| ATM | Abclonal | A19650 |
| Phospho-ATM (Ser1981) | Abclonal | AP1030 |
| H2AX | Cell Signaling Technology | 3636S |
| Phospho-H2AX (Ser139) | Abclonal | AP0687 |
| ARID1A | Cell Signaling Technology | 12354T |
| BRG1 | Cell Signaling Technology | 72182S |
| NBS1 | Abclonal | A21501 |
| 53BP1 | Abclonal | A5757 |
| ERCC1 | Proteintech | 66275-1-IG |
| ERCC2 | Abclonal | A14563 |
| ERCC3 | Abclonal | A12702 |
| ERCC6 | Abclonal | A18211 |
| ERCC8 | Abclonal | A6884 |
| AIM2 | Abclonal | A3356 |
| NLRP3 | Abclonal | A5652 |
| H3K9AC | EPIGENTEK | C10010-1 |
| H3K9Me3 | EPIGENTEK | A-4036-025 |
| H3K27Ac | EPIGENTEK | A-4708-100 |
| H3K27Me3 | EPIGENTEK | A-4036-100 |
| cGAS | Santa-Cruz | SC-515777 |
| STING | Proteintech | 19851-1-AP |
| CASPASE-1 | Proteintech | 22915-1-AP |
| β-Actin | Cell Signaling Technology | 12262S |
